# Longevity Factor Klotho and Resistance to Cognitive Deficits in Individuals with Parkinson’s Disease and in an α-Synuclein Mouse Model

**DOI:** 10.1523/JNEUROSCI.1904-25.2026

**Published:** 2026-03-31

**Authors:** Nijee S. Luthra, Luke W. Bonham, Arturo J. Moreno, Cana Park, Claire J. C. Huguenard, Samira Abdulai-Saiku, Shweta Gupta, Jonathan Lin, Lauren Broestl, Alexandre Bétourné, Rohan Sehgal, Dan Wang, Sylvain E. Lesné, Jill L. Ostrem, Jennifer S. Yokoyama, Dena B. Dubal

**Affiliations:** ^1^Department of Neurology, Weill Institute for Neurosciences, University of California, San Francisco, California 94158; ^2^Movement Disorders and Neuromodulation Center, University of California, San Francisco, California 94158; ^3^Department of Neuroscience and Institute for Translational Neuroscience, The University of Minnesota, Minneapolis, Minnesota 55455; ^4^Fein Memory and Aging Center, University of California, San Francisco, California 94158; ^5^Department of Radiology and Biomedical Imaging, University of California, San Francisco, California 94158; ^6^Bakar Aging Research Institute, University of California, San Francisco, California 94158

**Keywords:** aging, cognition, klotho, longevity, Parkinson’s disease, synuclein

## Abstract

Aging is the primary risk factor for Parkinson's disease (PD), and PD-related cognitive impairment remains a major unmet biomedical challenge. Klotho, a pleiotropic protein, extends lifespan and enhances cognition, but whether it confers resilience to cognitive impairments in PD is unclear. Here, we show that in humans, the KL-VS genetic variant of *KLOTHO*, linked to higher circulating klotho levels, associated with better executive cognition in individuals with PD across two independent cohorts. To test causality and explore mechanisms, we turned to mouse models. Transgenic elevation of klotho in a mouse model increased lifespan, improved synaptic and cognitive, but not motor, functions in mice, and decreased steady-state α-synuclein (α-syn) levels in the brains of male mice expressing wild-type human α-syn. Complementary in vitro studies showed that klotho rescued α-syn–induced deficits in NMDAR-dependent signaling through GluN2B and augmented α-syn microglial-related uptake, suggesting a potential mechanism by which klotho counters PD-related toxicity. Together, these findings indicate that klotho can counteract cognitive deficits related to PD, possibly by modulating α-syn levels, and these findings may be relevant to new therapeutic pathways for PD.

## Significance Statement

Klotho is a longevity factor that improves cognition in old mice, Alzheimer's disease model mice, and old nonhuman primates, yet its role Parkinson’s disease (PD)-related cognitive impairment remains unclear. Here, we identified an association between a *KLOTHO* variant and executive cognitive function in PD, a cognitive domain preferentially affected by the disease. To investigate biological mechanisms of klotho, we leveraged an α-synuclein (α-syn) mouse model of PD and found that klotho decreased cognitive deficits, potentially by increasing synaptic plasticity and reducing α-syn levels. Together, these findings suggest that klotho modulates PD-related cognitive deficits and highlight klotho-based strategies as promising therapeutic pathways for cognitive impairment in PD and other α-synucleinopathies.

## Introduction

Parkinson’s disease (PD) is an insidious neurodegenerative condition of aging. While PD classically impairs motor functions, we now know it also erodes cognition. Cognitive impairments—and specifically executive dysfunction—cause significant disability, manifest early (Darweesh et al., 2017a,b), predict incident disease (Ross et al., 2012), and eventually affect most individuals with PD (Aarsland et al., 2017). No effective medical treatments exist for cognitive deficits in PD. In light of this, pathways that delay aging, the primary risk factor for PD (Calne and Langston, 1983; Bennett et al., 1996; Tanner and Goldman, 1996), or enhance cognition itself could provide valuable leads.

Klotho, a pleiotropic protein named after a Greek Fate, slows aging (Kurosu et al., 2005) and enhances cognitive function across species. Klotho improves cognition in young and old mice (Dubal et al., 2014; Dubal et al., 2015; Leon et al., 2017; Gupta et al., 2022; Castner et al., 2023), old nonhuman primates (Castner et al., 2023), and mouse models of Alzheimer’s disease (AD; Dubal et al., 2015; Zeng et al., 2019; Zhao et al., 2020). Klotho also extends lifespan (Kurosu et al., 2005; Dubal et al., 2014) and regulates diverse functions ranging from insulin (Kurosu et al., 2005) and fibroblast growth factor signaling (Urakawa et al., 2006) to platelets (Park et al., 2023) and autophagy (Fernandez et al., 2018). It enhances cognition, in part, by increasing synaptic plasticity through NMDA receptor-dependent mechanisms (Dubal et al., 2014; Dubal et al., 2015; Leon et al., 2017).

In humans, the *KLOTHO* variant KL-VS associates with increased circulating klotho levels in heterozygotes (Arking et al., 2002; Dubal et al., 2014; Yokoyama et al., 2017). Across many studies, though not all (Porter et al., 2019; Muller et al., 2021; Amin et al., 2022; Shibata et al., 2025), KL-VS heterozygosity links with longevity (Arking et al., 2002; Arking et al., 2005; Invidia et al., 2010) and better cognitive performance in aging populations (Dubal et al., 2014; Yokoyama et al., 2015; de Vries et al., 2017; Yokoyama et al., 2017) as well as individuals with AD or AD risk (Belloy et al., 2020; Driscoll et al., 2021; Neitzel et al., 2021; Chen et al., 2023), with notable effects on executive cognition (Dubal et al., 2014; Yokoyama et al., 2015; Driscoll et al., 2021). While reduced klotho levels have been reported in PD (Sancesario et al., 2021; Zimmermann et al., 2021; Zimmermann et al., 2024; Yalcin et al., 2025), the role of *KLOTHO*—and specifically KL-VS heterozygosity—in cognitive domains vulnerable in PD, such as executive functions, remains largely unexplored. Furthermore, in contrast to established genetic risk factors for cognitive impairment in PD (Morley et al., 2012; Mata et al., 2014; Liu et al., 2017), potential sources of genetic resilience, such as KL-VS, remain a knowledge gap.

The pathologic hallmark of PD and related α-synucleinopathies is the deposition α-synuclein (α-syn), a presynaptic neuronal protein involved in vesicular packaging and transport (Kahle et al., 2000; Sulzer and Edwards, 2019). Mutations involving α-syn cause PD and include overexpression of the wild-type human protein (Goedert, 2001). Transgenic mice expressing human wild-type α-syn (hSYN) recapitulate some key features of PD such as dysfunction in dopaminergic pathways (Lam et al., 2011), motor impairment (Fleming et al., 2004; Morris et al., 2011; Leon et al., 2017), and, importantly, deficits in learning, memory, and synaptic plasticity (Magen et al., 2012; Ferreira et al., 2017; Leon et al., 2017).

Here we show, in two independent human cohorts with PD, KL-VS heterozygotes exhibited enhanced executive function, a cognitive domain impaired early in PD and predictive of dementia. To investigate mechanisms linking klotho to PD, we examined transgenic klotho overexpression in male hSYN mice and found that klotho elevation extended lifespan and attenuated cognitive and synaptic—but not motor—deficits, possibly through NMDAR signaling. Klotho also reduced hippocampal α-syn levels, potentially through enhanced microglial-related uptake. Together, these findings indicate that klotho can counteract PD-related cognitive deficits.

## Materials and Methods

### Human cohorts

Human data used in the preparation of this article was obtained on 2021-05-23 from the Parkinson's Progression Markers Initiative (PPMI) database, RRID:SCR_006431 (for up-to-date information on the study, visit www.ppmi-info.org) and from the National Institute of Neurological Disorders and Stroke Parkinson's Disease Biomarker Program (PDBP) database (http://pdbp.ninds.nih.gov/).

The PPMI study is an ongoing, longitudinal, prospective, observational study that aims to identify biomarkers for progression of PD. PPMI methodology and up-to-date information on the study is available at www.ppmi-info.org. Briefly, this multicenter, international study includes drug-naive patients with early-stage idiopathic PD and healthy controls (HC). Subjects underwent extensive clinical assessments (motor, neuropsychiatric, and cognitive), imaging, and collection of biological samples. Detailed inclusion and exclusion criteria have been described (Parkinson Progression Marker, 2011). Participants with PD (1) were >30 years of age, (2) presented two of three cardinal symptoms of PD (bradykinesia, rigidity, or resting tremor); (3) had a diagnosis within 2 years prior to entering the study; (4) were untreated for PD on entering the study; and (5) had a deficit of dopamine transporter, as assessed by ^123^I-ioflupane dopamine transporter DaTscan imaging. HC enrolled were over 30 years old, matched for age and sex, demographically comparable, and free of a current or active neurological disorder, and had no detectable dopamine transporter deficit evidence of PD.

PDBP is an aggregation of studies, each with its own inclusion and exclusion criteria (Gwinn et al., 2017). We specifically focused on participants from Phase III Study of Isradipine as a Disease Modifying Agent in Parkinson's Disease, STEADY-PD3 (PDBP-SY). The main inclusion criteria for this cohort (1) were >30 years of age, (2) presented two of three cardinal symptoms of PD (bradykinesia, rigidity, or resting tremor); (3) had a diagnosis within 3 years prior to entering the study; and (4) were untreated for PD on entering the study (Biglan et al., 2017).

### Genotyping of human cohorts

**Figure 1.**
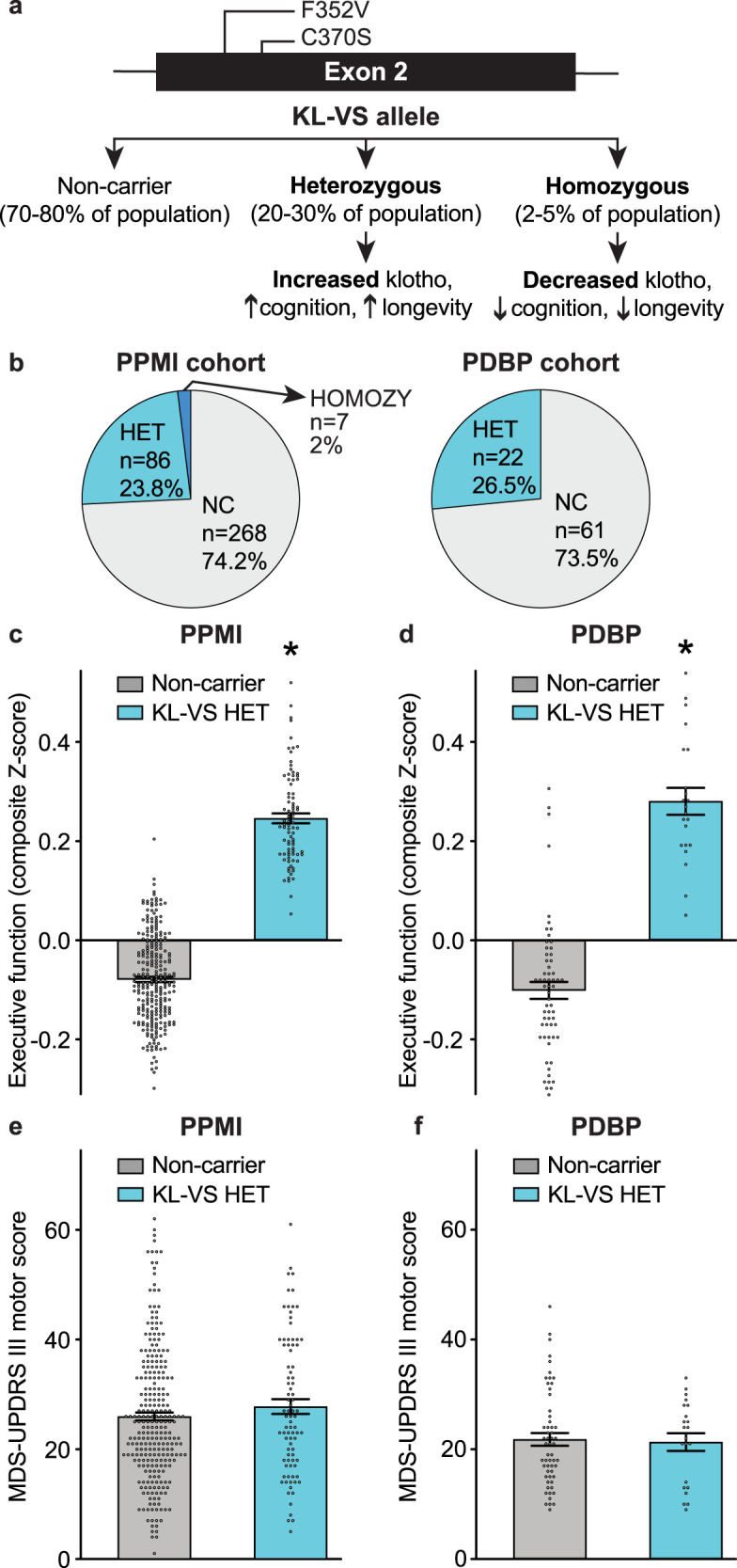
The KL-VS allele in heterozygosity associates with better cognitive but not motor performance in two independent PD cohorts. ***a***, Schematic representation of variants in human *KLOTHO* gene and its associations with cognition and longevity*.* Two variants in the *KLOTHO* gene, F352V and C370S, segregate together and form the protective KL-VS haplotype. In aging human populations, ∼70–80% are noncarriers, 20–30% are KL-VS heterozygotes, and 2–5% are KL-VS homozygotes. ***b***, Frequency of KL-VS noncarriers (NC), heterozygotes (HET), and homozygotes (HOMOZY) in meta-analysis of participants with PD within the PPMI and PDBP cohorts. Frequencies are similar to that described in the aging population. ***c,d***, Global executive function composite *Z*-scores in two independent cohorts of PD participants who are noncarriers or heterozygous carriers of the KL-VS allele. In each cohort, an individual composite score was standardized and scaled to reflect executive performance as a measure of the number of SDs from the global average of that cohort (global executive function composite *Z*-score). Higher scores indicate better cognitive performance. Data were analyzed by linear models, accounting for effects of age, sex, and education (for PPMI) and testing for effects due to KL-VS genotype. *APOE* ε4 and GBA carrier status had no significant effects (Table 2). ***c***, Global executive function composite *Z*-scores in PPMI cohort, with NC *n* = 268 and KL-VS HET *n* = 86, linear model: *p = 0.0167 (Table 2). ***d***, Global executive function composite *Z*-scores in PDBP cohort, with NC *n* = 61 and KL-VS HET *n* = 22; linear model, *p = 0.0320 (Table 2). ***e,f***, Motor function analyzed using the MDS-UPDRS III motor score, in two independent cohorts of PD participants who are NC versus KL-VS HET. Higher scores indicate worse motor performance. ***e***, Motor performance (MDS-UPDRS Part III motor score) in PPMI cohort, with NC *n* = 284 and KL-VS HET *n* = 90. ***f***, Motor performance (MDS-UPDRS Part III motor score) in PDBP cohort, with NC *n* = 57 and KL-VS HET *n* = 22. Bar graphs represent mean ± SEM.

Genotyping for the *KLOTHO* KL-VS variant was obtained from the PPMI and PDBP databases. All available biobanked DNA specimens from participants were genotyped using NeuroX. NeuroX was designed to include over 240,000 exonic variants, as well as over 24,000 variants specific to the study of neurodegenerative disease and provided information for the single nucleotide polymorphisms that compose the KL-VS variant*.* Two variants in the human *KLOTHO* gene, rs9536314 (F352V) and rs9527025 (C370S), segregate together and form the haplotype “KL-VS” (Fig. 1*a*; Arking et al., 2002). Based on whether or not they carried the KL-VS allele, subjects were identified as noncarriers, heterozygous (1 copy of KL-VS), or homozygous carriers (two copies of KL-VS). *APOE* ε4 and *GBA* pN370S carrier status were also available for participants in the PPMI and PDBP databases.

### Cognitive and motor function measures

In the current analysis, only noncarriers and KL-VS heterozygotes were analyzed; KL-VS homozygotes were excluded given the low number of subjects in this group*.* We chose to look at the baseline cognitive and motor scores in the PPMI and PDBP-SY database. In PPMI, cognition was assessed using a battery of neuropsychological tests as previously described (Caspell-Garcia et al., 2017) and which included Montreal Cognitive Assessment (MoCA) for global cognitive function, Hopkins Verbal Learning Test-Revised (HVLT-R) for memory, the Benton Judgment of Line Orientation 15-item (split-half) version for visuospatial function, the Symbol-Digit Modalities Test for processing speed-attention, and semantic fluency for verbal fluency. In PDBP-SY, only the MoCA testing was available. Test responses for executive/visuospatial, abstraction and phonemic verbal fluency from the MoCA were combined to assess executive cognition broadly and to decrease the risk of a Type 1 error by minimizing multiple comparisons. For each individual, test responses were standardized and then averaged resulting in a composite score. This score reflects the number of standard deviations above or below the global average (composite *Z*-score). Higher composite *Z*-scores reflect better executive function.

Motor disease severity was evaluated using the revised motor Unified Parkinson's Disease Rating Scale (UPDRS) published by the International Parkinson and Movement Disorder Society (MDS-UPDRS) and by the Hoehn and Yahr (H&Y) rating scale for stages of PD. To evaluate purely motor symptoms, only scores for MDS-UPDRS Part III were analyzed (Goetz et al., 2008).

### Statistical analysis of human data

The characteristics of participants from both PPMI and PDBP-SY cohorts are summarized using descriptive statistics. The significance of these differences was assessed using the *t* test for numeric variables and *χ*^2^ test for categorical variables (Table 1). For cognitive and motor measures, linear regression models were carried out in R (4.4.2). Total MoCA score and composite executive function *Z*-scores were analyzed independently in PPMI and PDBP-SY using multiple linear regression to evaluate the effects of the KL-VS genotype. In the PPMI cohort, all models covaried for age (years), sex (male or female), education (years), and APOE ε4 carrier status (carrier or noncarrier). In the PDBP-SY cohort, all models covaried for age (years), sex (male or female), and APOE ε4 carrier status (carrier or noncarrier). Education was not included in PDBP-SY analyses as the education level was unknown in most patients. As sensitivity analyses, all models were tested with carriers of GBA pN370S excluded. Similar multiple linear regression analysis was performed to analyze effects of the KL-VS genotype on the MDS-UDPRS III and H&Y stage for PPMI and PDBP-SY cohorts (Table 2).

**Table 1.** PPMI and PDBP-SY cohort characteristics

	PPMI			PDBP-SY		
	Noncarriers	KL-VS HET	*p* value	Noncarriers	KL-VS HET	*p* value
***n* (%)**	268 (74.2%)	86 (23.8%)	–	61 (73.5%)	22 (26.5%)	–
**Age in years (SD)**	61.32 (9.92)	62.12 (9.67)	0.51	62.77 (10.08)	62.64 (9.73)	0.96
**Education group**						
<12 years	13 (4.9%)	4 (4.7%)	0.41			
>16 years	153 (57.1%)	56 (65.1%)				
12–16 ears	102 (38.1%)	26 (30.2%)		6 (9.8)	3 (13.6)	0.93
Unknown	–			55 (90.2)	19 (86.4)	
**Sex (% male)**	180 (67.2%)	56 (65.1%)	0.83	43 (70.5%)	16 (72.3%)	1.00
**Race (% Caucasian)**	255 (95.1%)	85 (98.8%)	0.42	57 (93.4%)	22 (100.0%)	0.68
***APOE* ε4 Carrier**	72 (26.9%)	22 (25.6%)	0.93	11 (18.0%)	7 (31.8%)	0.30
**Years since symptom onset (SD)**	0.47 (0.55)	0.45 (0.51)	0.80	–	–	
**Years since diagnosis (SD)**	1.97 (2.20)	1.64 (1.10)	0.19	–	–	
**H&Y score**						
0	–	–		1 (1.8%)	0 (0%)	
1	124 (46.3%)	40 (46.5%)	1.00	22 (38.6%)	8 (36.4%)	0.80
2	144 (53.7%)	46 (53.5%)		34 (56.9%)	14 (63.6%)	
**UPDRS motor score (SD)**	20.49 (8.66)	21.26 (9.52)	0.49	23.91 (9.43)	18.86 (8.18)	0.03

Demographics were assessed for differences between groups in each cohort using *t* tests (numeric variables) or *χ*^2^ analyses (categorical variables). Standard deviation, SD.

**Table 2.** KL-VS heterozygotes in the PPMI and PDBP-SY cohorts show better executive function scores, but not better motor scores, compared with noncarriers

		Estimate	Std Error	*t* value	*p* value	Significance
**Global executive function composite *Z-*score *(GBA carriers included)***	**Cohort 1: PPMI**					
	Age	−0.0086	0.0053	−1.620	0.1061	–
	Sex (M)	0.0923	0.1108	0.832	0.4058	–
	Education	0.0634	0.0177	3.578	0.0004	***
	*APOE* ε4 carrier	0.0223	0.1181	0.189	0.8503	–
	Genotype, KL-VS	0.2921	0.1215	2.405	0.0167	*
	**Cohort 2: PDBP-SY**					
	Age	−0.0193	0.0060	−3.199	0.0020	**
	Sex (M)	−0.1131	0.1320	−0.857	0.3942	–
	*APOE* ε4 carrier	0.0857	0.1445	0.593	0.5551	–
	Genotype, KL-VS	0.2937	0.1345	2.184	0.0320	*
**Global executive function composite *Z-*score *(GBA carriers excluded)***	**Cohort 1: PPMI**					
	Age	−0.0093	0.0056	−1.642	0.1016	–
	Sex (M)	0.1009	0.1185	0.851	0.3954	–
	Education	0.0705	0.0183	3.859	0.0001	***
	*APOE* ε4 carrier	0.0309	0.1284	0.241	0.8100	–
	Genotype, KL-VS	0.2926	0.1300	2.250	0.0251	*
	**Cohort 2: PDBP-SY**					
	Age	−0.0198	0.0067	−2.948	0.0043	**
	Sex (M)	−0.1468	0.1406	−1.044	0.2999	–
	*APOE* ε4 carrier	0.0727	0.1495	0.486	0.6283	–
	Genotype, KL-VS	0.2845	0.1414	2.012	0.0480	*
**MoCA total score *(GBA carriers included)***	**Cohort 1: PPMI**					
	Age	−0.0426	0.0122	−3.500	0.0005	***
	Sex (M)	0.5100	0.2546	2.003	0.0459	*
	Education	0.0763	0.0407	1.874	0.0617	–
	*APOE* ε4 carrier	0.2491	0.2711	0.919	0.3588	–
	Genotype, KL-VS	−0.0073	0.2789	−0.026	0.9792	–
	**Cohort 2: PDBP-SY**					
	Age	−0.0489	0.0132	−3.700	0.0004	***
	Sex (M)	−0.0850	0.2885	−0.294	0.7692	–
	*APOE* ε4 carrier	0.2284	0.3159	0.723	0.4718	–
	Genotype, KL-VS	0.8386	0.2941	2.852	0.0056	**
**MoCA total score *(GBA carriers excluded)***	**Cohort 1: PPMI**					
	Age	−0.0448	0.0131	−3.423	0.0007	***
	Sex (M)	0.3673	0.2749	1.336	0.1825	–
	Education	0.0880	0.0423	2.079	0.0385	*
	*APOE* ε4 carrier	0.2863	0.2979	0.961	0.3372	–
	Genotype, KL-VS	0.0219	0.3015	0.073	0.9422	–
	**Cohort 2: PDBP-SY**					
	Age	−0.0443	0.0143	−3.096	0.0028	**
	Sex (M)	−0.0132	0.3002	−0.044	0.9651	–
	*APOE* ε4 carrier	0.2695	0.3192	0.844	0.4014	–
	Genotype, KL-VS	0.8159	0.3020	2.702	0.0086	**
**Motor function (MDS-UPDRS Part III Motor Score)**	**Cohort 1: PPMI**					
	Age	0.2259	0.0654	3.453	0.0006	***
	Sex (M)	−1.4960	1.3649	−1.096	0.2738	–
	*APOE* ε4 carrier	0.4837	1.4569	0.332	0.7401	–
	Genotype, KL-VS	1.6656	1.4929	1.116	0.2653	–
	**Cohort 2: PDBP-SY**					
	Age	0.0491	0.1023	0.480	0.6327	–
	Sex (M)	−0.4488	2.1550	−0.208	0.8356	–
	*APOE* ε4 carrier	−1.0736	2.3958	−0.448	0.6554	–
	Genotype, KL-VS	−0.3134	2.1907	−0.143	0.8866	–

KL-VS associated with increased performance in cognitive measures, but not motor function, with and without GBA pn370s carrier status. Comprehensive executive function was assessed through a composite score using visuospatial/executive, verbal fluency, and abstraction subscores from the MoCA. The linear statistical model includes age, sex, education (not available for PDBP-SY cohort), with and without *GBA* pN370S allele, and KL-VS genotype as predictors for cognitive and motor performance. *p < 0.05; **p < 0.01; ***p < 0.001.

Multiple linear regression models:

PPMI

CognitiveScore=β0+βAge+βSex+βEducation+βAPOEε4+βKL−VS,

PDBP-SY

CognitiveScore=β0+βAge+βSex+βAPOEε4+βKL−VS.

### Animals

Studies were conducted in a blinded manner in age-matched congenic C57BL/6J mice. Hemizygous transgenic mice expressing mouse klotho ubiquitously from the EF-1α promoter (Kuro-o et al., 1997) were crossed with hemizygous transgenic mice expressing wild-type human α-syn, *Thy-1-SYN* line 61, henceforward called hSYN (Rockenstein et al., 2002). All studies were conducted on age-matched littermates that included all four genotypes tested in-parallel: nontransgenic (NTG), klotho overexpression (KL), human α-syn (hSYN), and both human α-syn and klotho overexpression (hSYN/KL). Only male mice were used because the human α-syn transgene is inserted onto the X chromosome and randomly inactivates in females. Mice were kept on a 12 h light/dark cycle with *ad libitum* access to food (Picolab Rodent Diet20, Labdiet) and water. The studies were approved by the Institutional Animal Care and Use Committee of the University of California, San Francisco and conducted in compliance with NIH guidelines.

### Hindlimb clasp

The hindlimb clasp test was performed as described (Guyenet et al., 2010). Briefly, mice were held near the base of the tail and lifted upward away from surrounding objects. Mice hindlimbs were observed for 10 s and scored on a scale of 0–3: 0 indicated hindlimbs spread away from the core; 1 indicated retraction of the hindlimbs for <5 s; 2 indicated one of the hindlimbs retracted back for >5 s or both hindlimbs retracted for <5 s; and 3 indicated both hindlimbs retracted back for >5 s. This was repeated three times for each mouse, and the average score was calculated.

### Two-trial Y maze

The two-trial Y maze was performed as described (Leon et al., 2017). Briefly, following a 1 h habituation, mice were placed in a Y maze with spatial cues and allowed to explore one arm of the maze for a training session lasting 5 min, while the other arm was closed, establishing a familiar and novel arm, respectively. Sixteen hours after the training session, mice were allowed to explore both the novel and familiar arms. Distance traveled by the mice in each arm was recorded using the AnyMaze software.

### Open field

Before testing, mice were acclimated to the room for 30 min and then allowed to explore the open field for 10 min. In a clear plastic chamber (41 × 30 cm) with two rows of photobeams, total activity was detected by beam breaks and measured with an automated Flex-Field/Open Field Photobeam Activity System (San Diego Instruments).

### Balance beam

Balance beam testing was performed, with mice habituated in the testing room for 1 h. During pretraining, mice were placed in the center of the balance beam and allowed to walk to one end of the beam; the task was then repeated. Mice were then trained for 2 d on a medium-sized balance beam and allowed to walk across the length of the beam; each training day consisted of three trials. On Day 3, the mice were tested on a thinner beam for three trials, and their latency to cross and number of footslips were recorded.

### Rotarod

Rotarod testing was performed as described (Leon et al., 2017). Mice were placed in the testing room 1 h before training or testing. Mice were then placed on a fixed speed (16 rpm) rotarod for 5 min for a training session that consisted of three trials. The next day, mice were placed on an accelerating rotarod (4–40 rpm) for a morning and afternoon session that consisted of three trials each for a duration of 5 min per trial. The rotarod was cleaned with 70% ethanol between each group of mice. The latency for mice to fall off the rotarod was recorded and quantified for each genotype.

### Acute brain slices and field recordings

Acute brain slices and field recordings were carried out as described (Dubal et al., 2015; Leon et al., 2017) with minor modifications. Briefly, mice were anesthetized with isoflurane, and brains were quickly placed in ice-cold artificial cerebrospinal fluid (aCSF) containing the following (in mM): 124 NaCl, 2.8 KCl, 2 MgSO_4_, 10 glucose, 3.6 CaCl_2_, 1.25 NaH_2_PO_4_, 1.3 ascorbic acid, and 26 NaHCO_3_. For recordings containing Ro-25, the concentration of CaCl_2_ was lowered to 2.5 mM. The 300 µm sections were cut using a Leica Vibratome (VT1200). Slices were incubated at 32°C for 30 min and then allowed to recover at room temperature for 1 h. Slices were then transferred to an interface chamber with circulating oxygenated aCSF at 30°C and allowed to recover for 10 min before test stimulations. For field recordings, slices were placed on a MED64-Quad II multielectrode array (Alpha MED Scientific), which enables recording of four slices simultaneously. Field excitatory postsynaptic potentials (fEPSPs) were elicited and recorded via planar electrodes of the Quad II 2 × 8 Probe AL-MED-PG501A by aligning the electrodes and the dentate gyrus region of hippocampal slices. An input–output curve was performed at the beginning of each recording to determine the appropriate stimulation intensity. Test stimuli at 30–40% of maximal intensity were delivered at 0.05 Hz and a stable baseline of fEPSP of 15–20 min was established before long-term potentiation (LTP) induction. A 10 µM of bicuculline was added to the circulating bath to ensure robust induction of LTP in the dentate gyrus. LTP was induced using a theta-burst protocol comprised of three trains delivered every 20 s, each train containing 10 bursts at 5 Hz, each burst containing four pulses at 200 Hz. LTP was induced at 10 μA above test intensity and with the pulse width doubled. For Ro-25 experiments, once a stable baseline was established, 1 µM of Ro-25, diluted in DMSO, was placed in the bath and allowed to circulate for 20 min prior to LTP induction. Recordings and analysis were performed using the Med64 Mobius Software (Alpha MED Scientific).

### Protein extraction

Lysates were obtained from dissected mouse brains and homogenized in ice-cold lysis buffer 1× PBS, pH 7.4, 1 mM DTT, 0.5 mM EDTA, 0.5% Triton X-100, 1 mM phenylmethyl sulfonyl fluoride (PMSF), proteinase inhibitor (Roche), and phosphatase inhibitors 2 and 3 (Sigma-Aldrich). Samples were sonicated for 5 min, three times, and then centrifuged at 4°C for 10 min. Supernatant was collected and the concentration was determined by a BCA assay.

### Western blotting

For electrophoresis, 20 μg of protein was loaded into each well of a 4–12% gradient SDS–PAGE gel. Gels were transferred to nitrocellulose membranes and blocked with 5% BSA in TBS-T (0.05% Tween-20), or 5% BSA in TBS-T for phosphorylated antibodies, at room temperature for 1 h. Membranes were then immunoblotted with antibodies against total human α-syn (LB509, 1:1,000, Abcam), phosphorylated human α-syn (015-2519, 1:1,000, Wako Pure Chemical Industries), and klotho (KM2076, 1:1,000, Trans-Genic). Actin (MAB1501, 1:5,000, Millipore) and GAPDH (MAB374, 1:10,000, Millipore) were used as loading controls. Membranes were incubated at 4°C overnight, rinsed with TBS-T, incubated with secondary antibodies (LI-COR Biosciences), and then scanned using an Odyssey CLx (LI-COR Biosciences). Analysis of band intensity was performed using the ImageStudio software (LI-COR Biosciences).

### Quantitative reverse transcription (RT) PCR

Total RNA was extracted from dissected hippocampus with RNeasy Mini Kits (Qiagen). Total hippocampal RNA was reverse transcribed to generate cDNA (Applied Biosystems, Gene Amp PCR System 9700). Master mix for RT reaction contained MgCl_2_, dNTPs, Taqman RT Buffer, Hexamers, Poly dT primers, MultiScribe RT, and RNase Inhibitor. Primer sequences shown below were used.

Forward (5′→3′):

Mouse klotho: GTAGACGGGGTTGTAGCCAA
Human α-syn: CAACAGTGGCTGAGAAGACCA
Mouse α-syn: GGAGTGGTTCATGGAGTGACA
β-Actin: AGCAGGAGTACGATGAGTCC
GAPDH: GGGAAGCCCATCACCATCTT

Reverse (5′→3′):

Mouse klotho: GGTTATCTGAGGCCGGATGG
Human α-syn: GCTCCTTCTTCATTCTTGCCCA
Mouse α-syn: AGCTCCCTCCACTGTCTTCT
β-Actin: AGGGTGTAAAACGCAGCTCA
GAPDH: GCCTTCTCCATGGTGGTGAA

SYBR Green PCR Master Mix (Applied Biosystems) and ABI Prism 7900 HT sequence detection system (Applied Biosystems) were used. Each reaction contained Master Mix consisting of Nuclease Free H_2_O, Forward/Reverse Primers, and SYBR Green. Standard curves were generated with known standards of 3 ng/µl, 0.6 ng/µl, and 0.12 ng/µl. Dissociation curves (melting point analysis) were generated to test for accuracy of PCR products.

### RiboTag viral production and stereotaxic injection

RiboTag viral vector was based on AAV1 (AAV1- DIO-RiboTag; Sanz et al., 2015) and was a gift from Dr. G. Stanley McKnight (University of Washington). RiboTag AAV plasmids were packaged into AAV viral vectors, produced by Applied Biological Materials. Male (1.5–3 months of age) Syn1-cre/hSYN and Syn1-cre/hSYN/KL mice were anesthetized using isofluorane at 2–3% and placed in a stereotaxic frame. RiboTag AAV virus (5 μl per hemisphere) was stereotactically injected bilaterally into the dentate gyrus of the hippocampus using the following coordinates: AP, −2.1; ML, ±1.7; and DV, 1.9. Mice were allowed to wake up completely after surgery before being returned to their home cage. Mice were killed and underwent hippocampal dissection 4 weeks after AAV viral injections.

### RiboTag assay

RiboTag assay was performed as described (Sanz et al., 2019). The hippocampus was dissected and weighed before homogenization (2–5% wt/vol) in ice-cold homogenization buffer (50 mM Tris,100 mM KCl, 12 mM MgCl_2_, 1% Nonidet P-40, 1 mM DTT, 200 U/ml Promega RNasin, 1 mg/ml heparin, 100 µg/ml cycloheximide, Sigma protease inhibitor mixture, 1% sodium deoxycholate), pH 7.5. Samples were then centrifuged at 10,000 × *g* for 10 min at 4°C. Supernatants (800 µl) were incubated with 4 µl mouse monoclonal anti-HA antibody (H A.11, Covance) and rotated for 4 h at 4°C. A 200 µl of Protein A/G Magnetic Beads (Pierce) was washed once in 400 µl of homogenization buffer for 5 min at 4°C. The antibody-bound supernatants were then added directly to the washed beads and rotated overnight at 4°C. The following day, samples were placed on a magnet stand on ice, and supernatants were recovered before washing the pellets four times for 5 min in high salt buffer (50 mM Tris, 300 mM KCl, 12 mM MgCl_2_, 1% Nonidet P-40, 0.5 mM DTT, 100 µg/ml cycloheximide), pH 7.5. After washing, 350 µl Qiagen RLT buffer were added to the pellets. Total RNA was prepared according to manufacturer’s instructions using a RNeasy Mini kit (Qiagen) and quantified with a NanoDrop 1000 spectrophotometer (Thermo Fisher Scientific). qRT-PCR was performed on RNA to measure the expression of *klotho* and human *α-syn*.

### Microglial cell line treatments

The IMG Mouse Microglial cell line (Millipore; SCC134) was maintained in DMEM media supplemented with 10% FBS, 1% glutamine, and 1% penicillin/streptomycin. Cells were passaged every 4–5 d with 2.5% trypsin. All cell culture reagents were purchased from Invitrogen. Cells were plated at 1,000–5,000 cells per well for treatments, and all treatments were carried out on Day 3 and Day 6 in vitro in DMEM without FBS unless otherwise stated. Klotho was obtained at stock concentration of 50 µg and diluted to a working concentration of 250 ng/ml in cell media. Cells were plated at a density of 1,000 cells/well in a chamber slide (Ibidi; eight-well) and treated with 250 µg/ml klotho and 250 nM hSyn. EndoClear human α-syn (AS5555, AnaSpec) was reconstituted in PBS to a stock solution of 1 µg/µl, and a 250 nM working solution was prepared by further diluting in neurobasal media without B27 and immediately used; vehicle stock was prepared with equivalent DMSO volume in 1× PBS.

### Microglial cell line human α-syn ELISA

Enzyme-linked immunosorbent assays (ELISAs) were carried out as described (Wennstrom et al., 2012). Briefly, IMG cells were collected on Day 8 and homogenized in a lysis buffer containing protease and phosphate inhibitors. Following homogenization, samples were analyzed for human α-syn by ELISA following manufacturer's instructions (Thermo Fisher Scientific; KHB0061).

### Microglial cell line immunostaining

Treated IMG microglial cells were fixed and stained as described (Choi et al., 2020), with the primary antibody p62 (GP62-C, PROGEN, 1:250). The secondary antibody was guinea pig anti-rabbit Alexa Fluor 647 (Molecular Probes, 1:500). Cells were further stained with 3 nM DAPI (Sigma-Aldrich). Images were obtained at 100× using a spinning disk confocal microscope with a Nikon Qi2 camera.

### Statistical analyses of mouse studies and experimental design

Experimenters were blinded to the genotypes of mice. Statistical analyses were performed using GraphPad Prism (7.0) for *t* tests (two-tailed unless indicated otherwise), log-rank tests, and repeated-measures ANOVA. Exclusion criteria (>2 standard deviations above or below the mean) were defined a priori to ensure unbiased exclusion of outliers. Relative levels were determined using the background control genotype NTG, unless otherwise stated. R was used for mixed-model ANOVAs (nmle package), post hoc tests (p.adjust), and linear models (nmle package). Error bars represent the standard error of the mean (SEM), and null hypotheses were below or at a *p* value of 0.05.

### Data availability

Data are provided in source data files (Data S1).

### Code availability

No custom codes were used in this study.

## Results

### Human *KLOTHO* genetic variant KL-VS shows similar frequency in two independent cohorts of PD

We first determined whether a *KLOTHO* genetic variant associates with better cognitive functions using human subjects. Klotho has been linked to PD (Sancesario et al., 2021; Zimmermann et al., 2021; Zimmermann et al., 2024; Yalcin et al., 2025), but its specific relationship to relevant cognitive domains targeted in PD is unknown. To this end, we turned to two cohorts, the PPMI (Parkinson Progression Marker, 2011) and the PDBP. Within PDBP, we included participants who had been enrolled in STEADY-PD3 (Phase III Study of Isradipine as a Disease Modifying Agent in Parkinson's Disease) since this cohort's baseline characteristics matched those of the PPMI cohort, both containing idiopathic PD participants in early disease stage and not on dopaminergic medications, thus minimizing confounding variables. Baseline characteristics of PD participants genotyped for the *KLOTHO* gene variant, KL-VS, in both PPMI and STEADY-PD3 in PDBP (PDBP-SY) are shown in Table 1 (PPMI, *n* = 354; PDBP-SY, *n* = 83). KL-VS heterozygosity leads to higher klotho levels (Dubal et al., 2014; Yokoyama et al., 2017) and is associated with longevity (Arking et al., 2002; Arking et al., 2005; Invidia et al., 2010) and better cognitive functions in many (Dubal et al., 2014; Yokoyama et al., 2015; de Vries et al., 2017; Yokoyama et al., 2017), but not all (Mengel-From et al., 2016; Shibata et al., 2025), studies of aging populations.

The frequency of KL-VS in PPMI and PDBP-SY cohorts was similar to that described in normal aging populations (Arking et al., 2002; Dubal et al., 2014; Yokoyama et al., 2015; Fig. 1*a*). Within PPMI, 74.2% were noncarriers, 23.8% were heterozygous carriers, and 2% were homozygous carriers of KL-VS (Fig. 1*b*). Within PDBP-SY, there were 73.5% noncarriers and 26.5% heterozygous KL-VS carriers (Fig. 1*b*).

Since KL-VS homozygosity is rare and previously associated with lower klotho levels (Yokoyama et al., 2017), shorter lifespan, and decreased cognition (Arking et al., 2002; Arking et al., 2005; Deary et al., 2005; Yokoyama et al., 2017), this genotype was excluded from analysis.

Demographics and description of the PPMI and PDBP-SY cohorts are shown in Table 1. Mean age, race, sex distribution, and MoCA scores were similar between PPMI and PDBP-SY participants. Similar to the normal aging population (Farrer et al., 1997), the frequency of *APOE* ε4 was 26.6% in the PPMI cohort and 21.7% in the PDBP-SY cohort and did not differ between noncarriers and KL-VS heterozygotes.

### KL-VS heterozygosity associates with better executive cognition in PD

Cognitive dysfunction manifests early in PD (Darweesh et al., 2017a,b), predicts incident PD (Ross et al., 2012), and develops in the majority of individuals with the disease (Aarsland et al., 2017). Among the cognitive domains, executive functioning is impaired first and preferentially (Cronin-Golomb and Braun, 1997; Weintraub et al., 2005; Ross et al., 2012; Christopher et al., 2014) and predicts later development of dementia (Woods and Tröster, 2003; Janvin et al., 2005). Executive function includes measures such as working memory, task inhibition, and processing speed. Since KL-VS heterozygosity associates with better executive function in aging populations (Yokoyama et al., 2015; Yokoyama et al., 2017), including in individuals with high AD risk (Driscoll et al., 2021), we hypothesized that it would similarly associate with better executive function in PD.

Comprehensive executive function was assessed by creating a composite score using visuospatial/executive, verbal fluency, and abstraction subscores from the MoCA. Linear regression models were used to assess KL-VS genotype as a predictor of cognitive function, accounting for age, sex, education, and *APOE* ε4 carrier status as covariates. KL-VS heterozygosity associated with better executive cognition in both PPMI and PDBP-SY cohorts (Fig. 1*c*,*d*). In PDBP-SY, KL-VS carriers also had higher total MoCA scores (**p = 0.0056; Table 2). *APOE* ε4 carrier status did not contribute significant variance or influence any of the outcome measures (Table 2). Given previously reported links between cognition and PD risk variant *GBA* pN370S, we performed sensitivity analysis excluding participants with this variant, which did not change the observed significant differences (Table 2).

Given the association we observed between KL-VS heterozygosity and better executive function in PD patients, we next performed exploratory analyses testing whether KL-VS heterozygosity also associated with additional cognitive domains. We investigated MoCA subscores to measure memory (delayed recall), language (sentence repetition and fluency), and attention/information processing (serial 7s). All analyses were conducted using multiple regression covarying for age (years), sex (male or female), education (years), and *APOE* ε4 carrier status (carrier or noncarrier). We found a significant association between KL-VS heterozygosity and language fluency as measured by the number of words named as part of the MoCA (Table S1). There was no significant association between other memory or attention tasks and KL-VS heterozygosity. Since language fluency depends in part on executive function (Amunts et al., 2021), the increased fluency we observed in this data may be partially mediated by increased executive function.

### KL-VS heterozygosity does not associate with motor symptom severity in PD

We then examined whether KL-VS heterozygosity associates with better motor function. Measures of the MDS-UPDRS motor scale (Goetz et al., 2008) did not reveal differences between noncarriers and KL-VS heterozygotes in the PPMI cohort (Fig. 1*e*; Table 2). In the PDBP-SY, KL-VS was associated with improved MDS-UPDRS score (Table 1), but this did not remain significant once corrected for age, sex and *APOE* ε4 carrier status (Fig. 1*f*; Table 2). We also examined the relationship between KL-VS heterozygosity and the commonly used PD motor disease progression rating scale, H&Y. KL-VS did not associate with H&Y disease stage in PPMI or PDBP-SY cohorts (Table 1). Thus, KL-VS heterozygosity specifically associated with better executive cognitive function, but not measures of motor function, in individuals with PD.

### Klotho overexpression extends lifespan in hSYN mice

**Figure 2.**
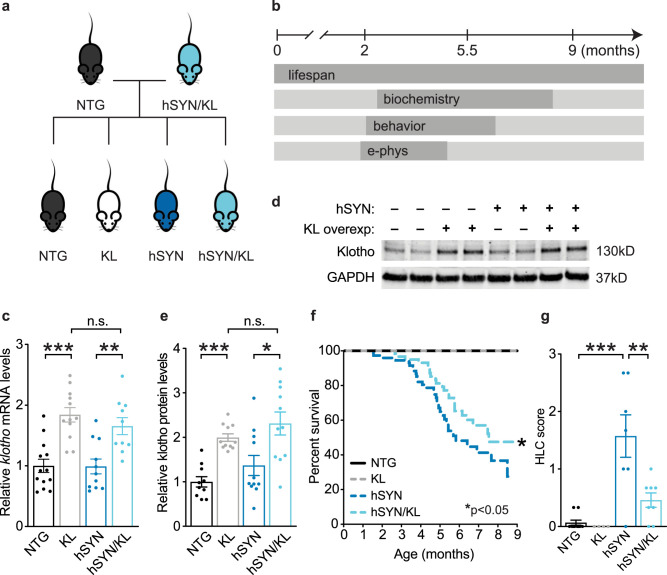
Transgenic overexpression of klotho extends lifespan and attenuates hindlimb clasping in human α-synuclein–expressing mice. ***a***, Breeding scheme for crossing klotho-overexpression (KL) and human α-syn–expressing mice (hSYN) with nontransgenic mice (NTG), electrophysiology (e-phys). ***b***, Diagram of the experimental timeline for testing multiple cohorts of mice across electrophysiology, behavioral, biochemical experiments, and lifespan. ***c***, Hippocampal klotho mRNA levels across experimental groups (NTG *n* = 13; KL *n* = 12; hSYN *n* = 11; and hSYN/KL *n* = 10 mice; age 4.2–7.9 months). Mean levels in NTG controls were arbitrarily defined as 1.0 and expressed relative to β-actin levels. Two-way ANOVA, KL effect ***p = 2.3350 × 10^−7^ (*F*_(1,42)_ = 37.95). Unpaired *t* tests (Bonferroni–Holm, B–H corrected): NTG versus KL ***p = 0.0001 (*t* = 5.218; df = 23), hSYN versus hSYN/KL **p = 0.0040 (*t* = 3.587; df = 19), KL/hSYN versus KL *p* = 0.3169 (*t* = 1.027; df = 20). ***d****,* Representative Western blot of hippocampal klotho and GAPDH levels. Images were captured from the same gel. ***e***, Hippocampal klotho protein levels determined by Western blot analysis (NTG *n* = 10; KL *n* = 11; hSYN *n* = 11; and hSYN/KL *n* = 11 mice; age 2.7–8.5 months). Mean levels in NTG controls were arbitrarily defined as 1.0 and expressed relative to GAPDH loading control. Two-way ANOVA, KL effect ***p = 8.3896 × 10^−6^ (*F*_(1,39)_ = 26.28). Unpaired *t* tests (B–H corrected): NTG versus KL ***p = 3.0000 × 10^−6^ (*t* = 7.011; df = 19), hSYN versus hSYN/KL *p = 0.0246 (*t* = 2.75; df = 20), NTG versus hSYN *p* = 0.1776 (*t* = 1.4; df = 19). ***f***, Kaplan–Meier curves showing the difference in survival among hSYN and hSYN/KL mice between weaning and 10 months of age (NTG *n* = 94; KL *n* = 52; hSYN *n* = 74; and hSYN/KL *n* = 62 mice); *χ*^2^ = 3.898; df = 1; *p = 0.0483 by Gehan–Breslow–Wilcoxon test; hSYN versus hSYN/KL. ***g***, Hindlimb clasp (HLC) scores (NTG *n* = 10, KL *n* = 4, hSYN *n* = 7, and hSYN/KL *n* = 8 mice, age 3.0–5.6 months). Two-way ANOVA: KL effect **p = 0.0080 (*F*_(1, 25)_ = 8.306), hSYN effect ***p = 6.2535 × 10^−5^ (*F*_(1, 25)_ = 23.04), and interaction effect *p = 0.0169 (*F*_(1, 25)_ = 6.551). Unpaired *t* tests (B–H corrected): NTG versus hSYN ***p = 0.0004 (*t* = 4.874; df = 15), hSYN versus hSYN/KL **p = 0.0099 (*t* = 3.019; df = 13). Bar graphs represent mean ± SEM.

To probe underlying mechanisms linking klotho to PD, we next examined transgenic klotho overexpression in a mouse model of α-synucleinopathy. To test whether klotho counteracts mortality and impairments in a mouse model of α-synucleinopathy, we first crossed hemizygous mice expressing wild-type human α-syn (hSYN) with hemizygous mice overexpressing klotho (KL) to produce double transgenic hSYN/KL mice. We then crossed hSYN/KL mice with nontransgenics (NTG) to produce four genotypes: NTG, KL, hSYN, and hSYN/KL mice (Fig. 2*a*). We used male mice since the human α-syn transgene is on the X chromosome and undergoes inactivation in female mice.

Multiple cohorts of mice underwent electrophysiology, behavioral tasks, biochemical experiments, and lifespan studies (Fig. 2*b*; Table S2). Transgenic klotho overexpression increased klotho mRNA (Fig. 2*c*) and protein levels (Fig. 2*d*,*e*) by approximately twofold in the hippocampus, a primary region of cognitive functions and klotho action (Dubal et al., 2014; Dubal et al., 2015), of KL mice with and without human α-syn.

Transgenic hSYN mice on a C57BL/6 background, as used here, display higher premature mortality (Coulombe et al., 2018) compared with that reported in the mixed C57BL/6-DBA2 line (Roshanbin et al., 2021). This is potentially related to network dysfunction and epileptic seizures previously observed in this model (Palop and Mucke, 2010; Morris et al., 2015). Interestingly, network dysfunctions are also observed in human synucleinopathies (McKeith et al., 2005; Andersson et al., 2008; Bonanni et al., 2008). Klotho overexpression extended lifespan in hSYN mice (Fig. 2*f*). Additionally, klotho decreased hSYN-induced abnormalities in the hindlimb clasp reflex (Fig. 2*g*), a gross test of central nervous system (CNS) function (Fernagut et al., 2004; Guyenet et al., 2010). Thus, transgenic klotho elevation improved survival and CNS integrity in hSYN mice.

### Klotho overexpression reduces cognitive and behavioral deficits in hSYN mice

**Figure 3.**
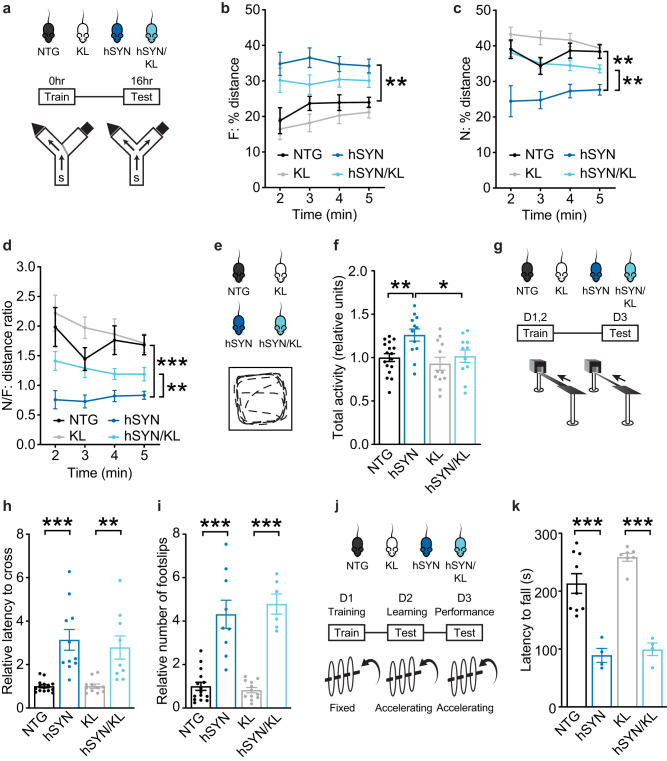
Klotho overexpression improves cognitive deficits, but not motor functions, in hSYN mice. ***a***, Diagram for testing spatial and working memory in the two-trial Y maze. Mice were exposed to a single arm during training. Mice were then tested 16 h later with both arms exposed; the previously exposed arm is designated as the familiar arm, and the newly exposed arm is designated as the novel arm. ***b***, Percentage distance traveled in the familiar arm (F) in the two-trial Y maze during 5 min of exploration (NTG = 7, KL = 13, hSYN = 9, and hSYN/KL = 11 mice, age 3.6–6.5 months). Two-way repeated–measure ANOVA, NTG versus hSYN effect **p = 0.0015 (*F*_(1,14)_ = 15.42). ***c***, Percentage distance traveled in the novel arm (N) in the two-trial Y maze during 5 min of exploration (NTG = 6, KL = 11, hSYN = 9, and hSYN/KL = 9 mice, age 3.6–6.5 months). Two-way repeated–measure ANOVA: hSYN versus hSYN/KL effect **p = 0.0024 (*F*_(1, 16)_ = 12.90), NTG versus hSYN effect **p = 0.0044 (*F*_(1,13)_ = 11.84). ***d***, Ratio of novel over familiar arm (N/F) percentage distance traveled in the two-trial Y maze during 5 min of exploration (NTG=6, KL = 10, hSYN = 9, and hSYN/KL = 9 mice, age 3.6–6.5 months). Two-way repeated–measure ANOVA: hSYN versus hSYN/KL effect **p = 0.0050 (*F*_(1,16)_ = 10.58), NTG versus hSYN effect ***p = 0.0005 (*F*_(1,13)_ = 21.54). ***e***, Diagram for testing activity in the open field, where mice explored freely for 10 min. ***f***, Total movements of mice relative to mean NTG control while exploring an open-field environment (NTG = 17, hSYN = 12, KL = 12, and hSYN/KL = 11 mice, age 2.1–4.4 months). Mean levels in NTG controls were arbitrarily defined as 1.0. Unpaired *t* tests (B–H corrected): NTG versus hSYN **p = 0.0090 (*t* = 3.267; df = 27), hSYN versus hSYN/KL *p = 0.0470 (*t* = 2.444; df = 21). ***g***, Diagram for testing motor coordination and fine movement using a balance beam. Mice were trained on Days 1 and 2 (three trials/day) and tested on Day 3 on a narrower beam (three trials). ***h***, Relative latency to cross balance beam across experimental groups (NTG *n* = 15, KL *n* = 11, hSYN *n* = 11, and hSYN/KL *n* = 9 mice, age 2.9–4.4 months). Mean levels in NTG controls arbitrarily defined as 1.0. Two-way ANOVA: hSYN effect ***p = 2.4886 × 10^−7^ (*F*_(1,42)_ = 37.71). Unpaired *t* tests (B–H corrected): NTG versus hSYN ***p = 0.0001 (*t* = 5.177; df = 24), KL versus hSYN/KL **p = 0.0020 (*t* = 3.618; df = 18). ***i***, Relative footslips on balance beam (NTG *n* = 15, KL *n* = 12, hSYN *n* = 9, and hSYN/KL *n* = 6 mice, age 2.8–4.4 months). Mean levels in NTG controls arbitrarily defined as 1.0. Two-way ANOVA: hSYN effect ***p = 2.3290 × 10^−12^ (*F*_(1,38)_ = 102.8). Unpaired *t* tests (B–H corrected): NTG versus hSYN ***p = 5.0000 × 10^−6^ (*t* = 6.032; df = 22), KL versus hSYN/KL ***p = 1.6000 × 10^−8^ (*t* = 10.92; df = 16). ***j***, Diagram for testing motor function on a rotarod; mice were trained on a fixed speed rotarod and underwent two sessions (three trials each) on an accelerating rotarod. Latency to fall (seconds), over the course of three trials in the learning session and three trials of the performance session, of the accelerating rotarod were recorded. ***k***, Latency to fall in seconds (s) during the performance session with an accelerating rotarod (NTG *n* = 9, KL *n* = 7, hSYN *n* = 4, and hSYN/KL *n* = 4 mice, age 3.6–4.6 months). Two-way ANOVA: hSYN effect ***p = 1.5707 × 10^−8^ (*F*_(1,20)_ = 82.39). Unpaired *t* tests (B–H corrected): NTG versus hSYN ***p = 0.0008 (*t* = 4.566; df = 11), KL versus hSYN/KL ***p = 7.3200 × 10^−7^ (*t* = 13.09; df = 9). Bar graphs represent mean ± SEM.

To determine whether transgenic klotho overexpression decreases cognitive deficits in hSYN mice, we assessed spatial and working memory in the two-trial Y maze (Fig. 3*a*). This task measures cognition through natural exploratory behavior and avoids the stress and motor demands of paradigms such as the Morris water maze. Since hSYN mice exhibit decreased motor function, the two-trial Y maze allows evaluation of cognitive performance without confounding motor impairments.

As expected (Leon et al., 2017), hSYN mice showed no preference for the novel arm, indicating impaired spatial and working memory (Fig. 3*b*–*d*; Fig. S1*a*–*c*). Klotho overexpression countered this cognitive impairment in hSYN mice (Fig. 3*c*,*d*). We then tested whether klotho attenuates other behavioral deficits. In the open-field test, which measures exploration and locomotor activity (Fig. 3*e*), hSYN mice were hyperactive, consistent with prior observations (Chesselet et al., 2012). Klotho blocked hyperactivity in hSYN mice (Fig. 3*f*). Thus, klotho overexpression improved cognitive and behavioral deficits in hSYN mice.

### Klotho overexpression does not improve motor function in hSYN mice

We next assessed whether klotho improved motor learning and function. We tested motor coordination and fine movement using a balance beam (Fig. 3*g*). As expected, latency to cross the balance beam and number of foot slips were increased in hSYN compared with NTG mice (Fig. 3*h*,*i*). Measures did not differ between hSYN mice with and without klotho overexpression (Fig. 3*h*,*i*). We next assessed motor learning and function using a rotarod (Fig. 3*j*). By the end of rotarod testing on Day 2, klotho overexpression did not improve motor function in hSYN mice (Fig. 3*k*). Thus, klotho did not improve motor functions in hSYN mice. Age did not significantly vary between groups for any of the behaviors performed (Fig. S2*a*–*f*).

### Klotho overexpression prevents hSYN-induced deficits in LTP

**Figure 4.**
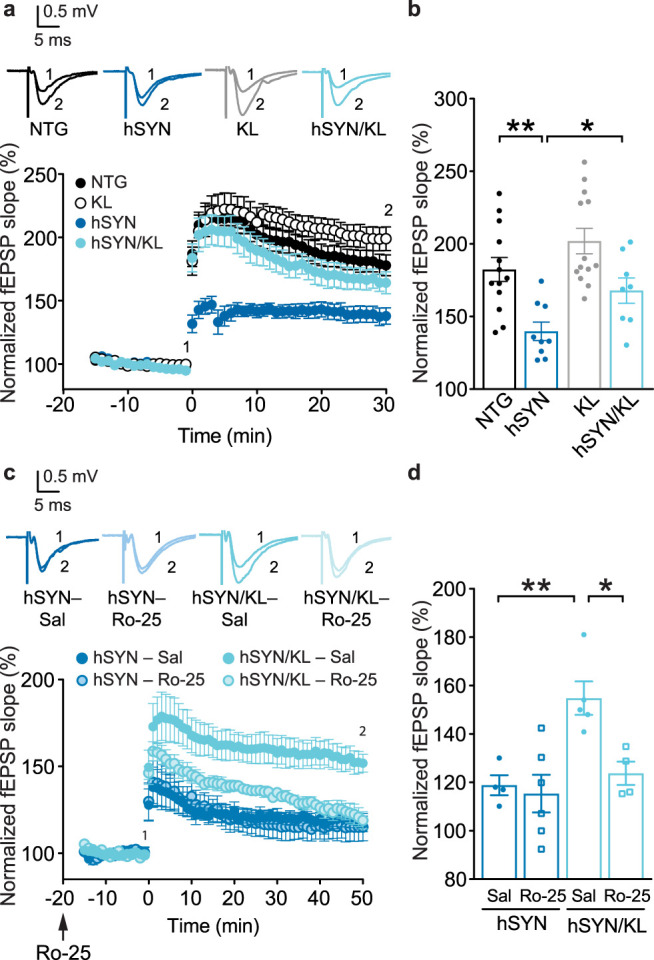
Klotho overexpression attenuates α-synuclein–induced synaptic deficits in the dentate gyrus, in a GluN2B-dependent manner. Field EPSPs were recorded in the dentate gyrus of 2- to 4-month-old mice. ***a***, LTP induction and decay were monitored for 30 min following theta-burst stimulation of the medial perforant pathway. The number of slices/number of mice: NTG 13/4, KL 13/5, hSYN 9/3, hSYN/KL 8/3. ***b***, Mean of the last 10 min of LTP recordings. Unpaired *t* tests (B–H corrected): NTG versus hSYN **p = 0.0024 (*t* = 3.77; df = 20), hSYN versus hSYN/KL *p = 0.0187 (*t* = 2.636; df = 15). ***c***, LTP induction and decay following theta-burst stimulation of the medial perforant pathway with and without Ro-25, a GluN2B inhibitor. Number of slices/number of mice: hSYN, saline (Sal) 4/3; hSYN, Ro-25 6/3; hSYN/KL, Sal 5/3; hSYN/KL, Ro-25 4/3. ***d***, Mean of the last 10 min of LTP recordings. Unpaired *t* tests (B–H corrected): hSYN-Sal versus hSYN/KL-Sal **p = 0.0126 (*t* = 4.171; df = 7), hSYN/KL Sal versus hSYN/KL Ro-25 *p = 0.0202 (*t* = 3.49; df = 7). Bar graphs represent mean ± SEM.

Since synaptic plasticity is a key substrate of learning and memory and hSYN mice show hippocampal-dependent cognitive deficits, we assessed whether human α-syn disrupts LTP and whether klotho attenuates this disruption. To this end, we performed multielectrode array recordings in the medial perforant pathway of the dentate gyrus of 2–4-month-old mice. We monitored the fEPSP following theta-burst–elicited LTP. Indeed, hSYN mice exhibited a robust deficit in LTP compared with NTG controls in the dentate gyrus, a deficit also observed with acute α-syn treatment in the CA1 region of wild-type rats (Diogenes et al., 2012; Fig. 4*a*,*b*). Overexpression of klotho blocked the α-syn–induced deficit (Fig. 4*a*,*b*), restoring synaptic plasticity to NTG levels. Thus, klotho probably improves cognition in hSYN mice, in part, by preventing synaptic dysfunction.

### Antagonizing GluN2B function abrogates klotho-mediated synaptic rescue

To begin to dissect how klotho blocks α-syn–induced deficits, we turned our attention to GluN2B, an NMDAR subunit key in learning and memory (Tang et al., 1999; Cao et al., 2007), and a synaptic target of both α-syn toxicity (Ferreira et al., 2017) and of klotho enhancement (Dubal et al., 2014; Dubal et al., 2015; Leon et al., 2017).

To assess if klotho-mediated rescue of α-syn–induced synaptic impairment requires GluN2B, we directly blocked GluN2B receptors by administering Ro-25, a highly specific GluN2B antagonist (Fischer et al., 1997), to hippocampal slices from hSYN mice. After incubation with Ro-25 or saline for 20 min, LTP was elicited using theta-burst stimulation. We first established a dose response in NTG slices (Fig. S3*a,b*). Because klotho preferentially engages GluN2B (Dubal et al., 2014; Dubal et al., 2015; Leon et al., 2017), we used the lowest dose of Ro-25 that enabled detection of a klotho effect but did not inhibit LTP in NTG slices (Fig. S3*c*,*d*). The low dose of Ro-25 completely blocked klotho-induced rescue in hSYN mice (Fig. 4*c*,*d*). We conclude that klotho overexpression rescues synaptic deficits in hSYN mice through GluN2B-dependent mechanisms of NMDA receptor signaling.

### Klotho overexpression decreases wild-type human α-syn at the protein but not mRNA level

**Figure 5.**
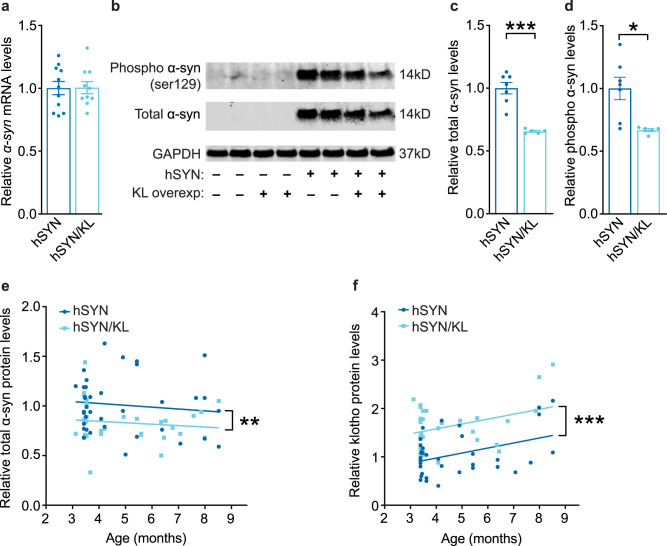
Klotho overexpression decreases human α-syn at the protein, but not mRNA, level. ***a***, Quantitation of hippocampal total mRNA levels of human *α-syn* in hSYN and hSYN/KL mice by RT-qPCR analysis (hSYN *n* = 11 and hSYN/KL *n* = 10 mice, age 4.2–7.9 months). Mean levels expressed relative to hSYN controls, arbitrarily defined as 1.0. GAPDH used as a loading control. ***b***, Representative Western blots of hippocampal levels of total and phosphorylated human α-syn protein in hSYN and hSYN/KL mice. GAPDH was used as a loading control. ***c***, Quantitation of Western blot signal of total human α-syn protein in hippocampal homogenates (hSYN *n* = 7 and hSYN/KL *n* = 5 mice, age 3.1–4.9 months) relative to hSYN mice; hSYN mean arbitrarily defined as 1.0. Unpaired *t* test: ***p = 0.0001 (*t* = 6.189; df = 10). ***d***, Quantitation of Western blot signal of phosphorylated human α-syn protein in hippocampal homogenates (hSYN *n* = 7 and hSYN/KL *n* = 5 mice, age 3.1–4.9 months) relative to hSYN mice; hSYN mean arbitrarily defined as 1.0. Unpaired *t* test: *p = 0.0117 (*t* = 3.076; df = 10). ***e***, Relative levels of total human α-syn protein plotted against age (hSYN *n* = 39 and hSYN/KL *n* = 34, age 3.1–8.5 months). Linear regression: difference in elevation (*y*-intercepts), **p = 0.0031 (*F* = 9.37; DFn = 1; DFd = 70). ***f***, Relative levels of klotho protein plotted against age (hSYN *n* = 37 and hSYN/KL *n* = 31 mice). Linear regression: difference in elevation (*y*-intercepts), ***p = 9.6538 × 10^−8^ (*F* = 36.01; DFn = 1; DFd = 65). Bar graphs represent mean ± SEM.

Since multiple deficits in hSYN mice depend on levels of human α-syn (Spillantini et al., 1998; Galvin et al., 1999), we assessed whether transgenic overexpression of klotho decreased α-syn. Consistent with what has previously been reported in the murine Thy-1–human α-syn model, we observed increased expression of α-syn mRNA and protein in the hippocampus (Rockenstein et al., 2002; Fig. 5*a*–*e*). While klotho overexpression in this model did not alter mRNA levels of *α-syn* (Fig. 5*a*), it surprisingly decreased both total and phosphorylated levels of human α-syn protein (Fig. 5*b*–*d*; Fig. S4*a*–*c*) in the hippocampus of the same mice. Notably, high-molecular-weight aggregates were absent at the ages studied in the hSYN model. These findings indicate that klotho overexpression decreases total α-syn protein levels independently of transcriptional changes or exogenous activation of the human α-syn promoter.

We also observed that total α-syn protein levels in hSYN/KL mice were consistently lower across the 3–9 months age range, while klotho remained elevated (Fig. 5*e*,*f*). This suggests that klotho does not delay the onset of α-syn burden but rather maintains a lower level across the lifespan measured.

### Klotho does not decrease translation of *α-syn* mRNA

**Figure 6.**
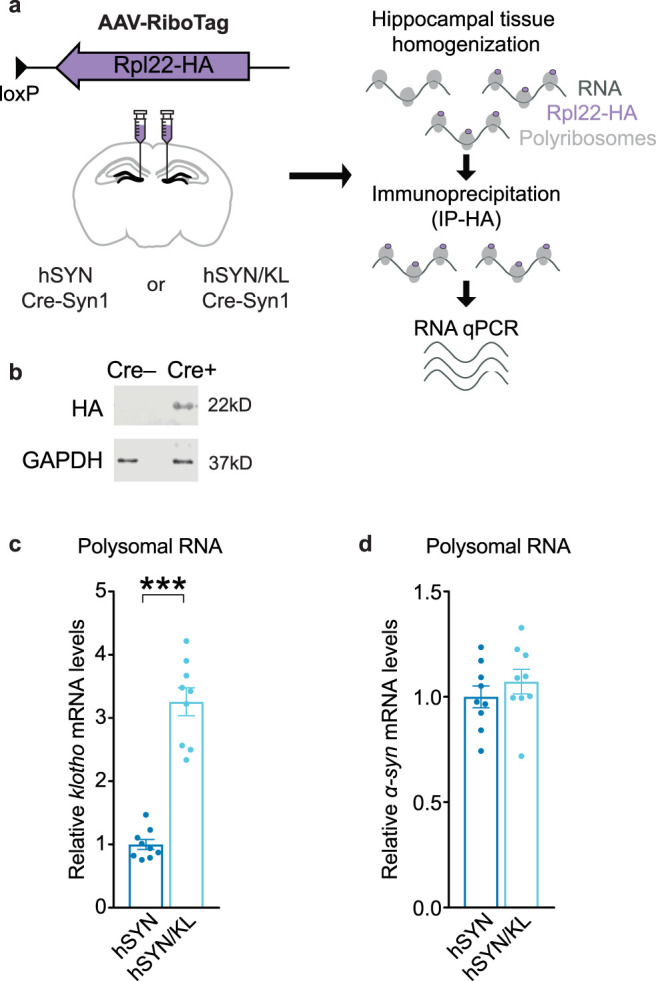
Klotho does not alter ribosomal translation of α*-synuclein* mRNA. ***a***, Diagram of the AAV-RiboTag approach. An AAV-RiboTag viral vector was injected into the dentate gyrus of Cre-Syn1 hSYN or Cre-SYN1 hSYN/KL mice. In the presence of Cre-recombinase, Rpl22-HA is switched on and expressed in Syn1-positive cells, where it labels actively translating ribosomes. HA immunoprecipitation (IP) enriches polysomes, and the associated RNA is isolated and analyzed by qRT-PCR. ***b***, Representative Western blots of expression levels of Rpl22-HA in the absence or presence of Cre-Syn1. ***c***, Quantification of hippocampal polysomal mRNA levels of *Klotho* mRNA by RT-qPCR analysis (hSYN *n*= 9, hSYN/KL *n* = 9 mice, age 2.5–4.0 months). hSYN mean levels arbitrarily defined as 1.0. *Actin* used as a loading control. Unpaired *t* test: ***p = 2.2084 × 10^−8^ (*t* = 9.663; df = 16). ***d***, Quantification of hippocampal polysomal mRNA levels of *α-syn* mRNA by RT-qPCR analysis (hSYN *n* = 9, hSYN/KL *n* = 9 mice, age 2.5–4.0 months). hSYN mean levels arbitrarily defined as 1.0. *Actin* used as a loading control.

We next examined whether klotho decreased translation of *α-syn* mRNA, thereby decreasing its protein levels. To probe this, we used an AAV-RiboTag system in hSYN mice with and without klotho overexpression to assess if klotho decreases RNA translation (Fig. 6*a*,*b*). As expected, transgenic klotho overexpression increased translating *klotho* mRNA levels associated with the ribosome (Fig. 6*c*). In contrast, klotho overexpression did not alter *α-syn* translation (Fig. 6*d*). These studies indicate that klotho did not decrease hippocampal α-syn levels by decreasing its mRNA translation.

### Klotho enhances uptake of α-syn in a microglial cell line

**Figure 7.**
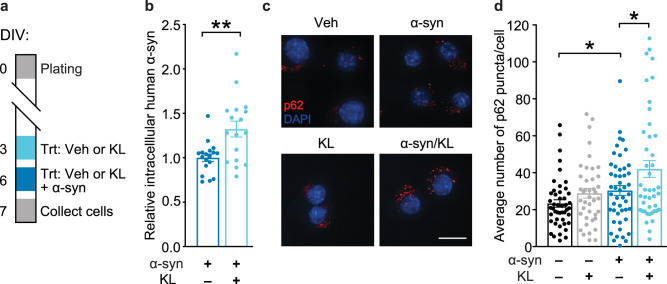
Klotho treatment increases α-synuclein uptake and p62 induction in a microglial cell line. ***a***, Schematic of microglial cell line culture and treatment paradigm. Cells were treated with Vehicle or Klotho (KL) on Day 3 in vitro (DIV 3) and again on DIV 6, with or without the addition of α-syn, and harvested on DIV 7. ***b***, Quantification of human α-syn protein levels from the cell lysates following Vehicle or Klotho treatment with or without α-syn (α-syn *n* = 18; α-syn/KL *n* = 17 samples from three independent experiments). Unpaired *t* test: **p = 0.0022 (*t* = 3.329; df = 33). ***c***, Representative immunocytochemistry images of p62 expression in cells following Vehicle or Klotho treatment with or without α-syn. Scale bar, 10 µm. ***d***, Quantification of the average number of p62 puncta per cells following Vehicle or Klotho treatment with or without α-syn (Veh *n* = 46, KL *n* = 41, α-syn *n* = 48, and α-syn/KL *n* = 43 cells from two independent experiments). Two-way ANOVA; KL effect **p = 0.0059 (*F*_(1,174)_ = 7.779), α-syn effect **p = 0.0013 (*F*_(1,174)_ = 10.69). Unpaired *t* tests: Veh versus α-syn *p = 0.0397 (*t* = 2.087; df = 92), α-syn versus α-syn/KL *p = 0.0258 (*t* = 2.268; df = 89). Bar graphs represent mean ± SEM.

To assess potential mechanisms by which klotho might influence turnover of α-syn, we turned to autophagy, a key protein degradation pathway. Microglia clear α-syn from the brain through selective autophagy (Choi et al., 2020), and klotho stimulates autophagy (Fernandez et al., 2018). We thus tested whether klotho enhances uptake of α-syn in a microglial cell line. Cells were treated with vehicle or klotho in the presence or absence of recombinant human α-syn (Fig. 7*a*). Klotho increased cellular uptake of human α-syn (Fig. 7*b*).

We then tested whether klotho stimulates the α-syn–selective p-62 autophagy pathway. As a multifaceted protein involved in autophagy, p62 is required for the assembly of α-syn to undergo clearance (Choi et al., 2020). As expected (Choi et al., 2020), we found that recombinant human α-syn induced p62 puncta (Fig. 7*c*,*d*). Klotho further increased p62 in α-syn–treated cells (Fig. 7*c*,*d*), suggesting enhanced microglial uptake and degradation of α-syn.

## Discussion

Our cognitive data in humans combined with behavioral, electrophysiological, and biochemical experiments in transgenic mice suggest that klotho can protect against PD-related cognitive deficits. In human studies, KL-VS heterozygosity, linked to higher circulating klotho levels, associated with better cognitive, but not motor, functions in early PD. To test causality, we examined mouse models and found that klotho overexpression increased survival and decreased cognitive, behavioral, and synaptic deficits induced by human α-syn, potentially through NMDAR-dependent mechanisms. Klotho overexpression also decreased total steady-state levels of the human α-syn protein in the hippocampus, potentially by increasing microglial-related α-syn uptake. Taken together, these data support klotho as a potential therapeutic factor to counteract cognitive dysfunction in neurodegenerative diseases like PD.

We found that in humans, genetic variation in *KLOTHO* associated with better cognitive, but not motor, functions in two independent cohorts of drug-naïve PD patients in early disease stages. Carrying one KL-VS allele leads to higher circulating klotho levels (Dubal et al., 2014; Yokoyama et al., 2017). In many studies, but not all (Porter et al., 2019; Muller et al., 2021; Amin et al., 2022; Shibata et al., 2025), KL-VS heterozygosity associates with longevity (Arking et al., 2002; Arking et al., 2005; Invidia et al., 2010) and better cognitive function in aging populations (Dubal et al., 2014; Yokoyama et al., 2015; de Vries et al., 2017; Yokoyama et al., 2017) and in those with AD (Neitzel et al., 2021; Chen et al., 2023) or AD risk (Driscoll et al., 2021). Since cognition sharply decreases with age in PD, it is interesting to speculate that KL-VS heterozygotes may be biologically younger than noncarriers and thus show better cognitive functions. It is important to note that blood or CSF levels of the klotho protein may be more closely associated with cognition or biomarkers, compared with *KLOTHO* genetic variants, as observed in an AD cohort (Grontvedt et al., 2022).

When measuring cognitive function in PD, task performance can be influenced by dopaminergic medications (Lange et al., 1995; Shohamy et al., 2005; Cools, 2006; Prescott et al., 2009; Gul and Yousaf, 2019) and disease stage, since pathology evolves (Litvan et al., 2011; Myers et al., 2021). To minimize these effects, we focused on PPMI and PDBP-SY cohorts, which included participants untreated with dopaminergic therapy and at early disease stages (within 2–3 years of diagnosis). In both cohorts, KL-VS heterozygosity was associated with better executive function, which is impaired early in PD and predictive of future disease (Ross et al., 2012; Kalbe et al., 2016; Darweesh et al., 2017a,b).

Our findings show that the link between KL-VS heterozygosity and better cognitive function extends to PD and may inform personalized genomics (Dubal and Yokoyama, 2020). The association between klotho and executive function is particularly intriguing since executive dysfunction in PD strongly predicts progression to dementia, and strategies enhancing executive function may modify cognitive decline. Prior studies show that in healthy aging, higher klotho levels associate with larger volumes of the dorsolateral prefrontal cortex, a key executive function region (Yokoyama et al., 2015). Future studies may explore whether klotho mediates cognitive function in PD through structural or connectivity changes.

Notably, the lack of *KLOTHO* associations with motor impairments in PD suggests neural specificity of klotho for cognitive neural networks, consistent with our mouse studies. Zimmermann et al. (2021) reported that low CSF klotho levels associated with greater motor impairment (MDS-UPDRS III and H&Y stage) in PD (Zimmermann et al., 2021). They also found reduced CSF klotho in PD compared with age-matched controls and that KL-VS heterozygous carriers had higher CSF klotho levels than noncarriers, further implicating klotho in PD. Although they did not observe a positive association between KL-VS and duration free from cognitive impairment, their subsequent study showed higher CSF klotho levels associated with later onset of cognitive impairment, including in *GBA1* carriers (Zimmermann et al., 2021; Zimmermann et al., 2024). One explanation may be their use of a single cutoff (MoCA ≤25) to define cognitive impairment. While commonly used for PD-MCI, the total MoCA score alone may lack sufficient sensitivity and specificity (Uysal-Cantürk et al., 2018). Given the limitations in cognitive assessment in PD, evaluating genetic effects across multiple clinical and pathological markers, particularly executive function, and replicating findings across cohorts remain important.

Furthermore, Zimmermann et al. (2021) examined more advanced disease stages of PD; we speculate that higher klotho levels may provide neuroprotection via cognitive reserve, delaying symptoms through greater tolerance to pathology. However, beyond a pathological threshold, cognitive decline may accelerate (Stern et al., 1999; Hall et al., 2007; Stern, 2009). Thus, klotho may increase buffering capacity against proteinopathies such as α-synucleinopathy in early disease stages.

Turning to our mouse findings, we leveraged a transgenic α-synucleinopathy model to explore causal and mechanistic explanations for the cognitive associations observed in humans. Lifelong transgenic klotho overexpression extended lifespan of hSYN mice. The premature mortality of hSYN mice on a pure C57Bl/6J background may relate to aberrant network excitability and seizures (Morris et al., 2015). Klotho overexpression may extend lifespan by preventing neural network dysfunction (Dubal et al., 2015), engaging longevity pathways involving autophagy and insulin signaling (Kurosu et al., 2005; Fernandez et al., 2018). α-Syn may drive cognitive deficits by activating aging-induced pathways that increase vulnerability to impaired proteostasis, energy metabolism, and mitochondrial function (López-Otín et al., 2023). As a longevity protein, klotho may intersect with aging mechanisms to promote resilience against α-syn toxicity.

In humans, hippocampal α-syn pathology is associated with dementia in PD, without frank neuronal loss; it is also present without frank dementia (Hall et al., 2014). Since spatial learning deficits are detectable before the onset of mild cognitive impairments (Schneider et al., 2017), these findings suggest spatial learning is an early and sensitive domain affected in PD, potentially preceding broader cognitive decline. This supports the relevance of our spatial memory assessments in modeling PD-related cognitive vulnerability while acknowledging limitations in extrapolating mouse behavior to human cognition.

Klotho is expressed in hippocampal neurons in humans and mice (Dias et al., 2021), and overexpression in hSYN mice blocked cognitive, behavioral, and synaptic deficits. Combined with evidence that klotho overexpression improves cognitive and synaptic functions in adult (Dubal et al., 2014; Leon et al., 2017), aging (Leon et al., 2017), and model AD mice (Dubal et al., 2015; Zeng et al., 2019; Zhao et al., 2020), these findings suggest klotho-mediated benefits are not limited to α-syn toxicity. Klotho may broadly enhance synaptic and cognitive reserve, increasing resilience against age- and disease-related pathophysiologies.

Blockade of GluN2B with low-dose Ro-25 did not alter synaptic plasticity of NTG or hSYN mice but completely abrogated klotho-mediated rescue of LTP in hSYN mice exposed to chronic α-syn toxicity. These electrophysiology findings suggest klotho counters α-syn toxicity by favorably modulating NMDAR function. GluN2B dysfunction contributes to synaptic impairments caused by acute, oligomeric α-syn (Ferreira et al., 2017). This raises questions about the specific role of GluN2B dysfunction in α-syn toxicity and whether it differs between acute and chronic exposure.

Transgenic klotho overexpression decreased total α-syn protein levels independent of transcriptional or translational changes and without exogenous activation of the human α-syn promoter. Klotho-mediated decreases in α-syn occurred with lifelong transgenic overexpression but not after a single acute treatment (Leon et al., 2017). One potential mechanism is enhanced degradation through increased autophagy (Fernandez et al., 2018), which can decrease α-syn levels (Spencer et al., 2009). We show that klotho enhances uptake of human α-syn and elevates p62, a key autophagy protein, in a microglial cell line. Whether inhibiting autophagy blocks klotho-mediated α-syn reduction remains unknown.

Elevated α-syn levels, arising from diverse etiologies (Goedert, 2001; Singleton et al., 2003; Theuns and Van Broeckhoven, 2008), contribute to PD, parkinsonian syndromes, and mixed proteinopathies observed across neurodegenerative diseases including AD (Kayed et al., 2020). Thus, approaches that lower α-syn, such as klotho-mediated pathways, may offer therapeutic potential for several neurodegenerative disorders.

Our findings in mice and humans suggest that increasing klotho levels could inform new therapies targeting cognitive impairment in PD and other synucleinopathies.

### Limitations

Although these findings identify klotho as a potential therapeutic factor that attenuates PD-related cognitive impairment and reduces α-syn levels, several limitations should be noted. Human studies need further assessment across geographically and demographically diverse populations. Mechanistic studies relied in the hSYN mouse model, which captures key aspects of α-synucleinopathy but does not reflect the full complexity of human PD, including varied genetic backgrounds, environmental factors, or extensive nigrostriatal dopaminergic degeneration. Thus, the model may be more relevant to early-midstage α-syn–driven mechanisms. Our analysis focused on hippocampal-dependent deficits and did not address other regions implicated in PD-related cognitive decline, including the prefrontal cortex. Since mouse motor measures used may not detect subtle or circuit-specific improvements with klotho, future studies with alternate assays may further define motor effects. As with all preclinical studies, extrapolating mouse behavioral phenotypes to human cognition has limitations. Finally, although clinical cohorts included both sexes, mouse experiments were limited to males because the human α-syn transgene is on the X chromosome and randomly inactivates in females.

## References

[B1] Aarsland D, Creese B, Politis M, Chaudhuri KR, Ffytche DH, Weintraub D, Ballard C (2017) Cognitive decline in Parkinson disease. Nat Rev Neurol 13:217–231. 10.1038/nrneurol.2017.27 28257128 PMC5643027

[B2] Amin HA, Cordell HJ, Martin-Ruiz C, Robinson L, Kirkwood T, Blakemore AI, Drenos F (2022) No evidence that genetic variation at the klotho locus is associated with longevity in caucasians from the Newcastle 85+ study and the UK biobank. J Gerontol A Biol Sci Med Sci 77:457–461. 10.1093/gerona/glab361 34893828 PMC8893196

[B3] Amunts J, Camilleri JA, Eickhoff SB, Patil KR, Heim S, von Polier GG, Weis S (2021) Comprehensive verbal fluency features predict executive function performance. Sci Rep 11:6929. 10.1038/s41598-021-85981-1 33767208 PMC7994566

[B4] Andersson M, Hansson O, Minthon L, Rosen I, Londos E (2008) Electroencephalogram variability in dementia with Lewy bodies, Alzheimer's disease and controls. Dement Geriatr Cogn Disord 26:284–290. 10.1159/000160962 18841014

[B5] Arking DE, et al. (2002) Association of human aging with a functional variant of klotho. Proc Natl Acad Sci U S A 99:856–861. 10.1073/pnas.022484299 11792841 PMC117395

[B6] Arking DE, Atzmon G, Arking A, Barzilai N, Dietz HC (2005) Association between a functional variant of the KLOTHO gene and high-density lipoprotein cholesterol, blood pressure, stroke, and longevity. Circ Res 96:412–418. 10.1161/01.RES.0000157171.04054.30 15677572

[B7] Belloy ME, Napolioni V, Han SS, Le Guen Y, Greicius MD, Alzheimer's Disease Neuroimaging, I (2020) Association of klotho-VS heterozygosity with risk of Alzheimer disease in individuals who carry APOE4. JAMA Neurol 77:849–862. 10.1001/jamaneurol.2020.0414 32282020 PMC7154955

[B8] Bennett DA, Beckett LA, Murray AM, Shannon KM, Goetz CG, Pilgrim DM, Evans DA (1996) Prevalence of parkinsonian signs and associated mortality in a community population of older people. N Engl J Med 334:71–76. 10.1056/NEJM199601113340202 8531961

[B9] Biglan KM, et al. (2017) A novel design of a phase III trial of isradipine in early Parkinson disease (STEADY-PD III). Ann Clin Transl Neurol 4:360–368. 10.1002/acn3.412 28589163 PMC5454402

[B10] Bonanni L, Thomas A, Tiraboschi P, Perfetti B, Varanese S, Onofrj M (2008) EEG comparisons in early Alzheimer's disease, dementia with Lewy bodies and Parkinson's disease with dementia patients with a 2-year follow-up. Brain 131:690–705. 10.1093/brain/awm322 18202105

[B11] Calne DB, Langston JW (1983) Aetiology of Parkinson's disease. Lancet 2:1457–1459. 10.1016/s0140-6736(83)90802-4 6140548

[B12] Cao X, Cui Z, Feng R, Tang YP, Qin Z, Mei B, Tsien JZ (2007) Maintenance of superior learning and memory function in NR2B transgenic mice during ageing. Eur J Neurosci 25:1815–1822. 10.1111/j.1460-9568.2007.05431.x 17432968

[B13] Caspell-Garcia C, et al. (2017) Multiple modality biomarker prediction of cognitive impairment in prospectively followed de novo Parkinson disease. PLoS One 12:e0175674. 10.1371/journal.pone.0175674 28520803 PMC5435130

[B14] Castner SA, et al. (2023) Longevity factor klotho enhances cognition in aged nonhuman primates. Nat Aging 3:931–937. 10.1038/s43587-023-00441-x 37400721 PMC10432271

[B15] Chen XR, Shao Y, Sadowski MJ, On Behalf Of The Alzheimer's Disease Neuroimaging, I (2023) Interaction between KLOTHO-VS heterozygosity and APOE epsilon4 allele predicts rate of cognitive decline in late-onset Alzheimer's disease. Genes (Basel) 14:917. 10.3390/genes14040917 37107675 PMC10137709

[B16] Chesselet MF, Richter F, Zhu C, Magen I, Watson MB, Subramaniam SR (2012) A progressive mouse model of Parkinson's disease: the Thy1-aSyn (“line 61”) mice. Neurotherapeutics 9:297–314. 10.1007/s13311-012-0104-2 22350713 PMC3337020

[B17] Choi I, Zhang Y, Seegobin SP, Pruvost M, Wang Q, Purtell K, Zhang B, Yue Z (2020) Microglia clear neuron-released alpha-synuclein via selective autophagy and prevent neurodegeneration. Nat Commun 11:1386. 10.1038/s41467-020-15119-w 32170061 PMC7069981

[B18] Christopher L, et al. (2014) Combined insular and striatal dopamine dysfunction are associated with executive deficits in Parkinson's disease with mild cognitive impairment. Brain 137:565–575. 10.1093/brain/awt337 24334314 PMC4454524

[B19] Cools R (2006) Dopaminergic modulation of cognitive function-implications for L-DOPA treatment in Parkinson's disease. Neurosci Biobehav Rev 30:1–23. 10.1016/j.neubiorev.2005.03.024 15935475

[B20] Coulombe K, Kerdiles O, Tremblay C, Emond V, Lebel M, Boulianne A-S, Plourde M, Cicchetti F, Calon F (2018) Impact of DHA intake in a mouse model of synucleinopathy. Exp Neurol 301:39–49. 10.1016/j.expneurol.2017.12.002 29229294

[B21] Cronin-Golomb A, Braun AE (1997) Visuospatial dysfunction and problem solving in Parkinson's disease. Neuropsychology 11:44–52. 10.1037//0894-4105.11.1.44 9055268

[B22] Darweesh SKL, Verlinden VJ, Stricker BH, Hofman A, Koudstaal PJ, Ikram MA (2017a) Trajectories of prediagnostic functioning in Parkinson's disease. Brain 140:429–441. 10.1093/brain/aww291 28082300

[B23] Darweesh SKL, Wolters FJ, Postuma RB, Stricker BH, Hofman A, Koudstaal PJ, Ikram MK, Ikram MA (2017b) Association between poor cognitive functioning and risk of incident Parkinsonism: the Rotterdam study. JAMA Neurol 74:1431–1438. 10.1001/jamaneurol.2017.2248 28973176 PMC5822187

[B24] Deary IJ, Harris SE, Fox HC, Hayward C, Wright AF, Starr JM, Whalley LJ (2005) KLOTHO genotype and cognitive ability in childhood and old age in the same individuals. Neurosci Lett 378:22–27. 10.1016/j.neulet.2004.12.005 15763166

[B25] de Vries CF, Staff RT, Harris SE, Chapko D, Williams DS, Reichert P, Ahearn T, McNeil CJ, Whalley LJ, Murray AD (2017) Klotho, APOEepsilon4, cognitive ability, brain size, atrophy, and survival: a study in the Aberdeen birth cohort of 1936. Neurobiol Aging 55:91–98. 10.1016/j.neurobiolaging.2017.02.019 28431289

[B26] Dias GP, et al. (2021) Intermittent fasting enhances long-term memory consolidation, adult hippocampal neurogenesis, and expression of longevity gene klotho. Mol Psychiatry 26:6365–6379. 10.1038/s41380-021-01102-4 34031536 PMC8760057

[B27] Diogenes MJ, et al. (2012) Extracellular alpha-synuclein oligomers modulate synaptic transmission and impair LTP via NMDA-receptor activation. J Neurosci 32:11750–11762. 10.1523/JNEUROSCI.0234-12.2012 22915117 PMC6703775

[B28] Driscoll I, et al. (2021) Age-related tau burden and cognitive deficits are attenuated in KLOTHO KL-VS heterozygotes. J Alzheimers Dis 79:1297–1305. 10.3233/JAD-200944 33427737 PMC9472657

[B29] Dubal DB, et al. (2014) Life extension factor klotho enhances cognition. Cell Rep 7:1065–1076. 10.1016/j.celrep.2014.03.076 24813892 PMC4176932

[B30] Dubal DB, et al. (2015) Life extension factor klotho prevents mortality and enhances cognition in hAPP transgenic mice. J Neurosci 35:2358–2371. 10.1523/JNEUROSCI.5791-12.2015 25673831 PMC4323521

[B31] Dubal DB, Yokoyama JS (2020) Longevity gene KLOTHO and Alzheimer disease-a better fate for individuals who carry APOE epsilon4. JAMA Neurol 77:798. 10.1001/jamaneurol.2020.0112 32282012

[B32] Farrer LA, Cupples LA, Haines JL, Hyman B, Kukull WA, Mayeux R, Myers RH, Pericak-Vance MA, Risch N, van Duijn CM (1997) Effects of age, sex, and ethnicity on the association between apolipoprotein E genotype and Alzheimer disease. a meta-analysis. APOE and Alzheimer disease meta analysis consortium. JAMA 278:1349–1356. 10.1001/jama.1997.03550160069041 9343467

[B33] Fernagut PO, Diguet E, Bioulac B, Tison F (2004) MPTP potentiates 3-nitropropionic acid-induced striatal damage in mice: reference to striatonigral degeneration. Exp Neurol 185:47–62. 10.1016/j.expneurol.2003.09.014 14697318

[B34] Fernandez AF, et al. (2018) Disruption of the beclin 1-BCL2 autophagy regulatory complex promotes longevity in mice. Nature 558:136–140. 10.1038/s41586-018-0162-7 29849149 PMC5992097

[B35] Ferreira DG, et al. (2017) Alpha-synuclein interacts with PrP(C) to induce cognitive impairment through mGluR5 and NMDAR2B. Nat Neurosci 20:1569–1579. 10.1038/nn.4648 28945221

[B36] Fischer G, Mutel V, Trube G, Malherbe P, Kew JN, Mohacsi E, Heitz MP, Kemp JA (1997) Ro 25-6981, a highly potent and selective blocker of N-methyl-D-aspartate receptors containing the NR2B subunit. Characterization in vitro. J Pharmacol Exp Ther 283:1285–1292. 9400004

[B37] Fleming SM, Salcedo J, Fernagut PO, Rockenstein E, Masliah E, Levine MS, Chesselet MF (2004) Early and progressive sensorimotor anomalies in mice overexpressing wild-type human alpha-synuclein. J Neurosci 24:9434–9440. 10.1523/JNEUROSCI.3080-04.2004 15496679 PMC6730110

[B38] Galvin JE, Uryu K, Lee VM, Trojanowski JQ (1999) Axon pathology in Parkinson's disease and Lewy body dementia hippocampus contains alpha-, beta-, and gamma-synuclein. Proc Natl Acad Sci U S A 96:13450–13455. 10.1073/pnas.96.23.13450 10557341 PMC23968

[B39] Goedert M (2001) Alpha-synuclein and neurodegenerative diseases. Nat Rev Neurosci 2:492–501. 10.1038/35081564 11433374

[B40] Goetz CG, et al. (2008) Movement disorder society-sponsored revision of the unified Parkinson's disease rating scale (MDS-UPDRS): scale presentation and clinimetric testing results. Mov Disord 23:2129–2170. 10.1002/mds.22340 19025984

[B41] Grontvedt GR, et al. (2022) Association of klotho protein levels and KL-VS heterozygosity with Alzheimer disease and amyloid and Tau burden. JAMA Netw Open 5:e2243232. 10.1001/jamanetworkopen.2022.43232 36413367 PMC9682425

[B42] Gul A, Yousaf J (2019) Effect of levodopa on frontal-subcortical and posterior cortical functioning in patients with Parkinson's disease. Singapore Med J 60:414–417. 10.11622/smedj.2018116 30246215 PMC6717772

[B43] Gupta S, et al. (2022) KL1 domain of longevity factor klotho mimics the metabolome of cognitive stimulation and enhances cognition in young and aging mice. J Neurosci 42:4016–4025. 10.1523/JNEUROSCI.2458-21.2022 35428698 PMC9097772

[B44] Guyenet SJ, Furrer SA, Damian VM, Baughan TD, La Spada AR, Garden GA (2010) A simple composite phenotype scoring system for evaluating mouse models of cerebellar ataxia. J Vis Exp 1787. 10.3791/1787 PMC3121238 20495529

[B45] Gwinn K, et al. (2017) Parkinson's disease biomarkers: perspective from the NINDS Parkinson's disease biomarkers program. Biomark Med 11:451–473. 10.2217/bmm-2016-0370 28644039 PMC5619098

[B46] Hall CB, Derby C, LeValley A, Katz MJ, Verghese J, Lipton RB (2007) Education delays accelerated decline on a memory test in persons who develop dementia. Neurology 69:1657–1664. 10.1212/01.wnl.0000278163.82636.30 17954781

[B47] Hall H, Reyes S, Landeck N, Bye C, Leanza G, Double K, Thompson L, Halliday G, Kirik D (2014) Hippocampal Lewy pathology and cholinergic dysfunction are associated with dementia in Parkinson's disease. Brain 137:2493–2508. 10.1093/brain/awu193 25062696

[B48] Invidia L, Salvioli S, Altilia S, Pierini M, Panourgia MP, Monti D, De Rango F, Passarino G, Franceschi C (2010) The frequency of klotho KL-VS polymorphism in a large Italian population, from young subjects to centenarians, suggests the presence of specific time windows for its effect. Biogerontology 11:67–73. 10.1007/s10522-009-9229-z 19421891

[B49] Janvin CC, Aarsland D, Larsen JP (2005) Cognitive predictors of dementia in Parkinson's disease: a community-based, 4-year longitudinal study. J Geriatr Psychiatry Neurol 18:149–154. 10.1177/0891988705277540 16100104

[B50] Kahle PJ, et al. (2000) Subcellular localization of wild-type and Parkinson's disease-associated mutant alpha-synuclein in human and transgenic mouse brain. J Neurosci 20:6365–6373. 10.1523/JNEUROSCI.20-17-06365.2000 10964942 PMC6772969

[B51] Kalbe E, et al. (2016) Subtypes of mild cognitive impairment in patients with Parkinson's disease: evidence from the LANDSCAPE study. J Neurol Neurosurg Psychiatry 87:1099–1105. 10.1136/jnnp-2016-313838 27401782

[B52] Kayed R, Dettmer U, Lesne SE (2020) Soluble endogenous oligomeric alpha-synuclein species in neurodegenerative diseases: expression, spreading, and cross-talk. J Parkinsons Dis 10:791–818. 10.3233/JPD-201965 32508330 PMC7458533

[B53] Kuro-o M, et al. (1997) Mutation of the mouse klotho gene leads to a syndrome resembling ageing. Nature 390:45–51. 10.1038/36285 9363890

[B54] Kurosu H, et al. (2005) Suppression of aging in mice by the hormone klotho. Science 309:1829–1833. 10.1126/science.1112766 16123266 PMC2536606

[B55] Lam HA, et al. (2011) Elevated tonic extracellular dopamine concentration and altered dopamine modulation of synaptic activity precede dopamine loss in the striatum of mice overexpressing human alpha-synuclein. J Neurosci Res 89:1091–1102. 10.1002/jnr.22611 21488084 PMC4755488

[B56] Lange KW, Paul GM, Naumann M, Gsell W (1995) Dopaminergic effects on cognitive performance in patients with Parkinson's disease. J Neural Transm Suppl 46:423–432. 8821078

[B57] Leon J, Moreno AJ, Garay BI, Chalkley RJ, Burlingame AL, Wang D, Dubal DB (2017) Peripheral elevation of a klotho fragment enhances brain function and resilience in young, aging, and alpha-synuclein transgenic mice. Cell Rep 20:1360–1371. 10.1016/j.celrep.2017.07.024 28793260 PMC5816951

[B58] Litvan I, Aarsland D, Adler CH, Goldman JG, Kulisevsky J, Mollenhauer B, Rodriguez-Oroz MC, Tröster AI, Weintraub D (2011) MDS task force on mild cognitive impairment in Parkinson's disease: critical review of PD-MCI. Mov Disord 26:1814–1824. 10.1002/mds.23823 21661055 PMC3181006

[B59] Liu G, et al. (2017) Prediction of cognition in Parkinson's disease with a clinical-genetic score: a longitudinal analysis of nine cohorts. Lancet Neurol 16:620–629. 10.1016/s1474-4422(17)30122-9 28629879 PMC5761650

[B60] López-Otín C, Blasco MA, Partridge L, Serrano M, Kroemer G (2023) Hallmarks of aging: an expanding universe. Cell 186:243–278. 10.1016/j.cell.2022.11.001 36599349

[B61] Magen I, et al. (2012) Cognitive deficits in a mouse model of pre-manifest Parkinson's disease. Eur J Neurosci 35:870–882. 10.1111/j.1460-9568.2012.08012.x 22356593 PMC3967873

[B62] Mata IF, et al. (2014) APOE, MAPT, and SNCA genes and cognitive performance in Parkinson disease. JAMA Neurol 71:1405–1412. 10.1001/jamaneurol.2014.1455 25178429 PMC4227942

[B63] McKeith IG, et al. (2005) Diagnosis and management of dementia with Lewy bodies: third report of the DLB consortium. Neurology 65:1863–1872. 10.1212/01.wnl.0000187889.17253.b1 16237129

[B64] Mengel-From J, Soerensen M, Nygaard M, McGue M, Christensen K, Christiansen L (2016) Genetic variants in KLOTHO associate with cognitive function in the oldest old group. J Gerontol A Biol Sci Med Sci 71:1151–1159. 10.1093/gerona/glv163 26405063 PMC4978356

[B65] Morley JF, et al. (2012) Genetic influences on cognitive decline in Parkinson's disease. Mov Disord 27:512–518. 10.1002/mds.24946 22344634 PMC3323737

[B66] Morris M, Koyama A, Masliah E, Mucke L (2011) Tau reduction does not prevent motor deficits in two mouse models of Parkinson's disease. PLoS One 6:e29257. 10.1371/journal.pone.0029257 22206005 PMC3242771

[B67] Morris M, et al. (2015) Network dysfunction in alpha-synuclein transgenic mice and human Lewy body dementia. Ann Clin Transl Neurol 2:1012–1028. 10.1002/acn3.257 26732627 PMC4693622

[B68] Muller BW, et al. (2021) Klotho KL-VS haplotype does not improve cognition in a population-based sample of adults age 55-87 years. Sci Rep 11:13852. 10.1038/s41598-021-93211-x 34226614 PMC8257625

[B69] Myers PS, Jackson JJ, Clover AK, Lessov-Schlaggar CN, Foster ER, Maiti B, Perlmutter JS, Campbell MC (2021) Distinct progression patterns across Parkinson disease clinical subtypes. Ann Clin Transl Neurol 8:1695–1708. 10.1002/acn3.51436 34310084 PMC8351397

[B70] Neitzel J, Franzmeier N, Rubinski A, Dichgans M, Brendel M, Alzheimer's Disease Neuroimaging, I, Malik R, Ewers M (2021) KL-VS heterozygosity is associated with lower amyloid-dependent tau accumulation and memory impairment in Alzheimer's disease. Nat Commun 12:3825. 10.1038/s41467-021-23755-z 34158479 PMC8219708

[B71] Palop JJ, Mucke L (2010) Amyloid-beta-induced neuronal dysfunction in Alzheimer's disease: from synapses toward neural networks. Nat Neurosci 13:812–818. 10.1038/nn.2583 20581818 PMC3072750

[B72] Park C, Hahn O, Gupta S, Moreno AJ, Marino F, Kedir B, Wang D, Villeda SA, Wyss-Coray T, Dubal DB (2023) Platelet factors are induced by longevity factor klotho and enhance cognition in young and aging mice. Nat Aging 3:1067–1078. 10.1038/s43587-023-00468-0 37587231 PMC10501899

[B73] Parkinson Progression Marker, I (2011) The Parkinson progression marker initiative (PPMI). Prog Neurobiol 95:629–635. 10.1016/j.pneurobio.2011.09.005 21930184 PMC9014725

[B74] Porter T, et al. (2019) Klotho allele status is not associated with abeta and APOE epsilon4-related cognitive decline in preclinical Alzheimer's disease. Neurobiol Aging 76:162–165. 10.1016/j.neurobiolaging.2018.12.014 30716541

[B75] Prescott IA, Dostrovsky JO, Moro E, Hodaie M, Lozano AM, Hutchison WD (2009) Levodopa enhances synaptic plasticity in the substantia nigra pars reticulata of Parkinson's disease patients. Brain 132:309–318. 10.1093/brain/awn322 19050033

[B76] Rockenstein E, Mallory M, Hashimoto M, Song D, Shults CW, Lang I, Masliah E (2002) Differential neuropathological alterations in transgenic mice expressing α-synuclein from the platelet-derived growth factor and Thy-1 promoters. J Neurosci Res 68:568–578. 10.1002/jnr.10231 12111846

[B77] Roshanbin S, Aniszewska A, Gumucio A, Masliah E, Erlandsson A, Bergstrom J, Ingelsson M, Ekmark-Lewen S (2021) Age-related increase of alpha-synuclein oligomers is associated with motor disturbances in L61 transgenic mice. Neurobiol Aging 101:207–220. 10.1016/j.neurobiolaging.2021.01.010 33639338 PMC9648497

[B78] Ross GW, Abbott RD, Petrovitch H, Tanner CM, White LR (2012) Pre-motor features of Parkinson's disease: the Honolulu-Asia aging study experience. Parkinsonism Relat Disord 18:S199–202. 10.1016/S1353-8020(11)70062-1 22166434

[B79] Sancesario GM, et al. (2021) Biofluids profile of α-Klotho in patients with Parkinson's disease. Parkinsonism Relat Disord 90:62–64. 10.1016/j.parkreldis.2021.08.004 34392132

[B80] Sanz E, Quintana A, Deem JD, Steiner RA, Palmiter RD, McKnight GS (2015) Fertility-regulating Kiss1 neurons arise from hypothalamic POMC-expressing progenitors. J Neurosci 35:5549–5556. 10.1523/JNEUROSCI.3614-14.2015 25855171 PMC4388920

[B81] Sanz E, Bean JC, Carey DP, Quintana A, McKnight GS (2019) Ribotag: ribosomal tagging strategy to analyze cell-type-specific mRNA expression in vivo. Curr Protoc Neurosci 88:e77. 10.1002/cpns.77 31216392 PMC6615552

[B82] Schneider CB, Linse K, Schonfeld R, Brown S, Koch R, Reichmann H, Leplow B, Storch A (2017) Spatial learning deficits in Parkinson's disease with and without mild cognitive impairment. Parkinsonism Relat Disord 36:83–88. 10.1016/j.parkreldis.2016.12.020 28027851

[B83] Shibata K, Chen C, Tai XY, Manohar SG, Husain M (2025) Impact of APOE, klotho, and sex on cognitive decline with aging. Proc Natl Acad Sci U S A 122:e2416042122. 10.1073/pnas.2416042122 39903109 PMC11831164

[B84] Shohamy D, Myers CE, Grossman S, Sage J, Gluck MA (2005) The role of dopamine in cognitive sequence learning: evidence from Parkinson's disease. Behav Brain Res 156:191–199. 10.1016/j.bbr.2004.05.023 15582105

[B85] Singleton AB, et al. (2003) Alpha-synuclein locus triplication causes Parkinson's disease. Science 302:841. 10.1126/science.1090278 14593171

[B86] Spencer B, Potkar R, Trejo M, Rockenstein E, Patrick C, Gindi R, Adame A, Wyss-Coray T, Masliah E (2009) Beclin 1 gene transfer activates autophagy and ameliorates the neurodegenerative pathology in alpha-synuclein models of Parkinson's and Lewy body diseases. J Neurosci 29:13578–13588. 10.1523/JNEUROSCI.4390-09.2009 19864570 PMC2812014

[B87] Spillantini MG, Crowther RA, Jakes R, Hasegawa M, Goedert M (1998) Alpha-synuclein in filamentous inclusions of Lewy bodies from Parkinson's disease and dementia with Lewy bodies. Proc Natl Acad Sci U S A 95:6469–6473. 10.1073/pnas.95.11.6469 9600990 PMC27806

[B88] Stern Y, Albert S, Tang MX, Tsai WY (1999) Rate of memory decline in AD is related to education and occupation: cognitive reserve? Neurology 53:1942–1947. 10.1212/wnl.53.9.1942 10599762

[B89] Stern Y (2009) Cognitive reserve. Neuropsychologia 47:2015–2028. 10.1016/j.neuropsychologia.2009.03.004 19467352 PMC2739591

[B90] Sulzer D, Edwards RH (2019) The physiological role of alpha-synuclein and its relationship to Parkinson's disease. J Neurochem 150:475–486. 10.1111/jnc.14810 31269263 PMC6707892

[B91] Tang YP, Shimizu E, Dube GR, Rampon C, Kerchner GA, Zhuo M, Liu G, Tsien JZ (1999) Genetic enhancement of learning and memory in mice. Nature 401:63–69. 10.1038/43432 10485705

[B92] Tanner CM, Goldman SM (1996) Epidemiology of Parkinson's disease. Neurol Clin 14:317–335. 10.1016/S0733-8619(05)70259-0 8827174 PMC7173037

[B93] Theuns J, Van Broeckhoven C (2008) Alpha-synuclein gene duplications in sporadic Parkinson disease. Neurology 70:7–9. 10.1212/01.wnl.0000295508.10258.6b 18166703

[B94] Urakawa I, Yamazaki Y, Shimada T, Iijima K, Hasegawa H, Okawa K, Fujita T, Fukumoto S, Yamashita T (2006) Klotho converts canonical FGF receptor into a specific receptor for FGF23. Nature 444:770–774. 10.1038/nature05315 17086194

[B95] Uysal-Cantürk P, Hanağası HA, Bilgiç B, Gürvit H, Emre M (2018) An assessment of movement disorder society task force diagnostic criteria for mild cognitive impairment in Parkinson's disease. Eur J Neurol 25:148–153. 10.1111/ene.13467 28941002

[B96] Weintraub D, Moberg PJ, Culbertson WC, Duda JE, Katz IR, Stern MB (2005) Dimensions of executive function in Parkinson's disease. Dement Geriatr Cogn Disord 20:140–144. 10.1159/000087043 16020942

[B97] Wennstrom M, Londos E, Minthon L, Nielsen HM (2012) Altered CSF orexin and alpha-synuclein levels in dementia patients. J Alzheimers Dis 29:125–132. 10.3233/JAD-2012-111655 22207004

[B98] Woods SP, Tröster AI (2003) Prodromal frontal/executive dysfunction predicts incident dementia in Parkinson's disease. J Int Neuropsychol Soc 9:17–24. 10.1017/s1355617703910022 12570354

[B99] Yalcin A, Gemci E, Yurumez B, Yilmaz R, Varli M, Atmis V, Akbostancı MC, Yazihan N (2025) Serum alpha klotho levels in Parkinson's disease. Neurol Sci 46:743–749. 10.1007/s10072-024-07809-w 39467935

[B100] Yokoyama JS, et al. (2015) Variation in longevity gene KLOTHO is associated with greater cortical volumes. Ann Clin Transl Neurol 2:215–230. 10.1002/acn3.161 25815349 PMC4369272

[B101] Yokoyama JS, et al. (2017) Systemic klotho is associated with KLOTHO variation and predicts intrinsic cortical connectivity in healthy human aging. Brain Imaging Behav 11:391–400. 10.1007/s11682-016-9598-2 27714549 PMC5382127

[B102] Zeng CY, Yang TT, Zhou HJ, Zhao Y, Kuang X, Duan W, Du JR (2019) Lentiviral vector-mediated overexpression of klotho in the brain improves Alzheimer's disease-like pathology and cognitive deficits in mice. Neurobiol Aging 78:18–28. 10.1016/j.neurobiolaging.2019.02.003 30851437

[B103] Zhao Y, Zeng CY, Li XH, Yang TT, Kuang X, Du JR (2020) Klotho overexpression improves amyloid-beta clearance and cognition in the APP/PS1 mouse model of Alzheimer's disease. Aging Cell 19:e13239. 10.1111/acel.13239 32964663 PMC7576297

[B104] Zimmermann M, et al. (2021) The longevity gene klotho and its cerebrospinal fluid protein profiles as a modifier for Parkinson´s disease. Eur J Neurol 28:1557–1565. 10.1111/ene.14733 33449400

[B105] Zimmermann M, et al. (2024) Association of elevated cerebrospinal fluid levels of the longevity protein α-klotho with a delayed onset of cognitive impairment in Parkinson's disease patients. Eur J Neurol 31:e16388. 10.1111/ene.16388 38946703 PMC11414814

